# Interplay between hippocampal TACR3 and systemic testosterone in regulating anxiety-associated synaptic plasticity

**DOI:** 10.1038/s41380-023-02361-z

**Published:** 2023-12-22

**Authors:** Magdalena Natalia Wojtas, Marta Diaz-González, Nadezhda Stavtseva, Yuval Shoam, Poonam Verma, Assaf Buberman, Inbar Izhak, Aria Geva, Roi Basch, Alberto Ouro, Lucia Perez-Benitez, Uri Levy, Erika Borcel, Ángel Nuñez, Cesar Venero, Noa Rotem-Dai, Isana Veksler-Lublinsky, Shira Knafo

**Affiliations:** 1 Department of Physiology and Cell Biology, The National Institute for Biotechnology in the Negev, and the School of Brain Sciences and Cognition, Ben-Gurion University of the Negev, Beer-Sheva, Israel; 2 Instituto Biofisika (UPV/EHU, CSIC), Departamento Biología Celular e Histología Facultad de Medicina y Enfermería, University of the Basque Country, Leioa, Spain; 3 Department of Psychobiology, Universidad Nacional de Educación a Distancia (UNED), Madrid, Spain; 4 Departamento de Anatomía, Histología y Neurociencia, Facultad de Medicina, Universidad Autonoma de Madrid, Madrid, Spain; 5 Department of Software and Information Systems Engineering, Ben-Gurion University of the Negev, Beer-Sheva, Israel; 6 Ikerbasque, Basque Foundation for Science, Bilbao, 48013 Spain; 7 Present Address: NeuroAging Group Laboratory (NEURAL), Clinical Neurosciences Research Laboratory (LINC), Health Research Institute of Santiago de Compostela (IDIS), Santiago de Compostela, Spain; 8 Present Address: Centro de investigación Biomédica en Red de Enfermedades Neurodegenerativas (CIBERNED), Instituto de Salud Carlos III, Madrid, Spain; 9 Present Address: Department of Clinical Neuroscience, Centre Hospitalier Universitaire Vaudois (CHUV), Lausanne, Switzerland

**Keywords:** Neuroscience, Psychiatric disorders

## Abstract

Tachykinin receptor 3 (TACR3) is a member of the tachykinin receptor family and falls within the rhodopsin subfamily. As a G protein-coupled receptor, it responds to neurokinin B (NKB), its high-affinity ligand. Dysfunctional TACR3 has been associated with pubertal failure and anxiety, yet the mechanisms underlying this remain unclear. Hence, we have investigated the relationship between TACR3 expression, anxiety, sex hormones, and synaptic plasticity in a rat model, which indicated that severe anxiety is linked to dampened TACR3 expression in the ventral hippocampus. TACR3 expression in female rats fluctuates during the estrous cycle, reflecting sensitivity to sex hormones. Indeed, in males, sexual development is associated with a substantial increase in hippocampal TACR3 expression, coinciding with elevated serum testosterone and a significant reduction in anxiety. TACR3 is predominantly expressed in the cell membrane, including the presynaptic compartment, and its modulation significantly influences synaptic activity. Inhibition of TACR3 activity provokes hyperactivation of CaMKII and enhanced AMPA receptor phosphorylation, associated with an increase in spine density. Using a multielectrode array, stronger cross-correlation of firing was evident among neurons following TACR3 inhibition, indicating enhanced connectivity. Deficient TACR3 activity in rats led to lower serum testosterone levels, as well as increased spine density and impaired long-term potentiation (LTP) in the dentate gyrus. Remarkably, aberrant expression of functional TACR3 in spines results in spine shrinkage and pruning, while expression of defective TACR3 increases spine density, size, and the magnitude of cross-correlation. The firing pattern in response to LTP induction was inadequate in neurons expressing defective TACR3, which could be rectified by treatment with testosterone. In conclusion, our study provides valuable insights into the intricate interplay between TACR3, sex hormones, anxiety, and synaptic plasticity. These findings highlight potential targets for therapeutic interventions to alleviate anxiety in individuals with TACR3 dysfunction and the implications of TACR3 in anxiety-related neural changes provide an avenue for future research in the field.

## Introduction

Human puberty initiates with the re-emergence of pulsatile Gonadotropin-releasing hormone (GnRH) secretion and the progressive activation of gonadal function [1, 2]. The failure to produce gonadal hormones defines hypogonadotropic hypogonadism, which is associated with reduced testosterone levels [3, 4]. Testosterone secretion is governed by neuroendocrine events in the hypothalamic-pituitary-gonadal (HPG) axis [5]. Indeed, neurons within the preoptic area of the hypothalamus release GnRH, which in turn stimulates the anterior pituitary to secrete luteinizing hormone and follicle-stimulating hormone [6, 7]. Testosterone’s initial role occurs during the prenatal phase, contributing to sex differentiation, the development of male genitalia, and various aspects of sexual imprinting [8].

Testosterone controls different psychological, sexological, relational, cognitive, and reproductive aspects of behavior, which may be significantly affected by a deficiency or decrease in testosterone. Low testosterone levels reduce sexual desire, produce erectile and female sexual dysfunction, deteriorate memory, and induce a loss of energy and poor concentration, as well as other symptoms [9–11]. Testosterone has a profound role in modulating mental health, having notable effects in severe psychiatric diseases like schizophrenia, mood disorders, and anxiety-related conditions [12]. It contributes to the complex psychological balance in men, affecting mood, behavior, self-perception, and overall quality of life, and it also plays a role in the manifestation of depressive symptoms, ranging from mild dysthymia to severe suicidal ideation [13]. Notably, the influence of testosterone extends to aspects of anxiety, aggression, and risk-taking behaviors, highlighting its pervasive role in a variety of mental disorders. Indeed, testosterone deficiency can exacerbate psychiatric symptoms [13, 14]. Significantly, studies involving testosterone supplementation also highlight its therapeutic potential, particularly in older men with hypogonadism [13], suggesting it could be used to potentially manage psychiatric disorders.

In non-syndromic normosmic congenital hypogonadotropic hypogonadism (CHH), a failure to produce gonadal hormones results in lower testosterone levels [15]. This disorder is caused by missense loss-of-function mutations in Tac3 and Tacr3, the genes encoding neurokinin B (TAC3) and its NK3R receptor (TACR3), respectively [16–18]. TACR3 is a G protein-coupled receptor widely distributed in the brain, particularly in the hypothalamus, pituitary gland, and hippocampus, and it is known to modulate gonadotropin release through its action on Kisspeptin-1 neurons [19, 20]. Downregulation of TACR3 was observed in the lateral habenula of mice showing anxiety-like behaviors, whereas TACR3 overexpression in the same area significantly reversed such anxiety-like behaviors [21]. Indeed, it is notable that the most common psychopathological conditions in young hypogonadal men are depression and anxiety [3, 22, 23]. Anxiety in these patients has been attributed to their low testosterone levels, and the psychological consequences of this [3], and testosterone therapy improves the symptoms of sexual dysfunction, depression, and anxiety in these patients [3, 22, 23]. The strong link between testosterone levels and anxiety has been well-established [24, 25], although the molecular pathways linking testosterone to anxiety are poorly understood.

This study focused on the intricate relationship between hippocampal TACR3 expression, plasticity-related signaling pathways, sex hormones, and anxiety-like behavior. The findings highlight the pivotal role of TACR3 as a critical mediator between testosterone levels and anxiety-like behavior. Notably, we demonstrate the modulatory effect of sex hormones on TACR3 expression and its reciprocal control over sex hormone levels and anxiety-like behavior. Furthermore, our results highlight the direct impact of altered TACR3 activity on neuronal connectivity and impaired long-term potentiation in hippocampal neurons, which can be rescued through testosterone treatment.

## Materials and methods

All the experiments carried out here were previously approved by the committees for ethical care and use of animals for experimentation at the Ben-Gurion University (b14764_30) and the University of the Basque Country (M20/2016/001; M20/2018/296; M20/2016/019). The experiments were all carried out in accordance with the guidelines of the European Community Council Directives (2010/63/EU).

### Animals

Experiments were conducted using adult Wistar rats weighing between 280 and 450 grams and aged 3–4 months, as well as rats ranging from embryonic day 18 (E18) to 30 days old for developmental studies. With the exception of a biochemical investigation focusing on TACR3 expression during the estrous cycle, all other experiments exclusively utilized male rats. Prior to the experimental phase, animals were acclimated for a minimum of 15 days in Plexiglas cages, housing five rats per cage unless specified otherwise. These rats were maintained under controlled environmental conditions, with a temperature of 21 ± 2 °C and a 12:12-h light-dark cycle (lighting from 07:00 to 19:00 h). Throughout the acclimation period, the rats had ad libitum access to food and water, which was restricted only during active experimentation.

### Blinding procedures

All behavioral, electrophysiological, and morphological experiments were conducted under blinded conditions to minimize bias. The investigator performing the assessments was unaware of the treatment groups or experimental conditions until the completion of data analysis.

### Randomization procedure

Animals were randomized into different experimental groups based on their weight to ensure an equitable distribution across all conditions. Random assignment was then carried out within each stratum to ensure that each experimental group had a comparable range and mean weight.

### Gene expression analysis

Gene expression was analyzed using RNA extracted from the ventral hippocampus of male rats displaying Severe (SA) or Moderate Anxiety (MA: Supplementary Fig. 1). Differentially expressed genes (DEGs) were defined as those with a absolute log-fold change between SA and MA anxiety above 1, and *P* value < 0.05. Gene ontology (GO) and KEGG enrichment analysis was performed using DAVID [26].

### Elevated plus-maze

**Fig. 1:**
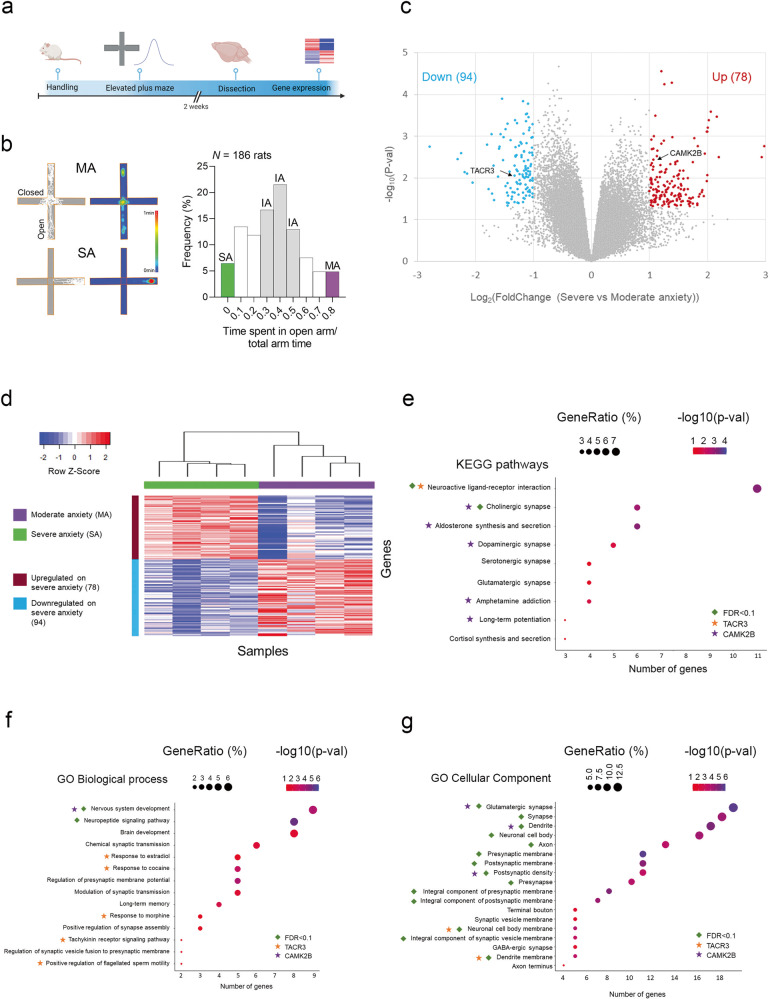
Analysis of hippocampal gene expression in rats with diverse anxiety-like behaviors. **a** Experimental design. Rats were categorized based on their performance in the elevated plus maze (EPM), and two weeks later, their hippocampus was extracted for gene expression analysis. **b** Classification of rats in the EPM. *Left*: Representative traces from the EPM showing the path (left) and color-coded time spent in each location of the maze (right) by rats categorized with moderate (MA) or severe anxiety (SA). *Right*: Frequency distribution of the EPM scores for all rats: the rats with extreme scores, indicated in color, were selected for gene expression analysis. **c** Volcano plot of the differential gene expression in MA and SA rats. Upregulated genes are shown in red, downregulated genes in blue, and non-significantly changed genes in gray, based on their statistical significance (-log10 *p*-value) and fold change (log2 fold change) values. **d** Hierarchical clustering was performed on eight samples (four with SA and four with MA) using Euclidean distances calculated from the expression of 172 differentially expressed genes (DEGs). The clustering analysis resulted in the formation of distinct clusters, and the colors in the heat map represent row-scaled expression values, with blue indicating weak expression and red indicating strong expression. Dot plots illustrating the enriched (**e**) KEGG pathways, (**f**) GO biological processes, and (**g**) GO cellular components associated with the DEGs. Each dot’s position on the x-axis represents the number of genes out of the 172 DEGs enriched for the corresponding term displayed on the y-axis. The dot’s size and color indicate the GeneRatio (proportion of DEGs within the pathway/process/component out of the 143 DEGs found in the DAVID database) and the level of significance, respectively. The terms are ordered based on the number of DEGs on the x-axis. Terms with an FDR (False Discovery Rate) < 0.1 or containing TACR3 or CAMK2B genes are marked [26] (For a comprehensive list of genes, see 10.5281/zenodo.8305270).

We assessed anxiety-related behavior with the elevated plus-maze (EPM) test. The maze has two open arms (measuring 45 × 10 cm) and two enclosed arms (measuring 45 × 10 × 50 cm) located opposite each other, and it is connected to a central platform (measuring 10 × 10 cm) elevated 65 cm above the floor. For juvenile rats, we used a smaller maze (arms dimensions: 35 × 5 × 40 cm). Each rat was placed in the central compartment facing one of the enclosed arms and allowed to freely explore the maze for 5 min. The rats’ movements were recorded with a video camera and analyzed with a computerized tracking system (AnyMaze, Stoelting) that registered their entry into an arm as soon as all four paws were in it. The time spent in the open and closed arms and in the central compartment was recorded. To quantify the anxiety-like behavior of the rats, we divided the time spent in the open arm by the total time in both arms. This value was used to classify rats as having moderate (MA), intermediate (IA) or severe (SA) anxiety based on their scores relative to the overall distribution of the scores from four experiments involving 186 rats (Fig. 1a, b). Specifically, MA rats had scores above the 90th percentile, SA rats had scores below the 10th percentile, and IA rats had scores between the 40th and 60th percentiles.

### In vivo testosterone/osanetant treatment and measurements

#### Testosterone treatment

Adult male Wistar rats (3 months old) were injected subcutaneously on five consecutive days with the vehicle alone (mineral oil) or with testosterone propionate (5 mg/kg/day: #86541-5 G Sigma-Aldrich, Fig. 3d). The hippocampus was then extracted and lysed by sonication, and TACR3 was analyzed in western blots.

#### Osanetant treatment

Animals were divided into two groups, control or osanetant treated (Sigma, SML0798), and the latter were administered osanetant (25 mM) prepared from a stock solution diluted in DMSO while the control animals were injected with the same volume of saline (vehicle). Before treatment, the stock solution was diluted in 0.9% sterile saline up to 1 ml for each animal according to their body mass. Osanetant was administered intraperitoneally (ip) at a dose of 5 mg/kg.

#### Blood collection and hormone assays

Blood samples (approximately 200 μl) were collected twice from the tail vein: before treatment and one day after the last treatment with osanetant or testosterone. Blood samples were centrifuged at 10,000 g for 5 min at 20 °C, and the serum retrieved was stored at –20 °C for further analyses.

#### Testosterone measurement

Following Testosterone treatment, serum testosterone was measured with the testosterone Parameter Assay Kit (R&D Systems, #KGE010) as indicated by the manufacturer. For osanetant treatment, serum testosterone was measured at the Endocrinology Lab of the Soroka Medical Center by competitive Immunoassay using direct chemiluminescent Technology on an ADVIA Centaur® XPT machine (SIEMENS). The threshold for detection was 0.07 ng/mL.

#### Implantation of in vivo mini-osmotic pumps

Anesthetization of male Wistar rats (age: 3 months) was achieved using 2.5% isoflurane. Intracerebroventricular (i.c.v.) delivery cannulas from Alzet’s brain infusion kit II were surgically implanted using a stereotaxic frame (KOPF Instruments). The implantation was carried out at specific coordinates relative to the bregma: AP, −0.8 mm; ML, +1.6 mm; and DV, −4.0 mm. Osmotic minipumps (Alzet; model #2004) were loaded with either 100 nM of Osanetant (Sigma-Aldrich; SML0798) or a control vehicle (sterile 0.9% NaCl of medical grade). These pumps were pre-equilibrated in 0.9% NaCl solution at 37 °C for 48 h. Subsequently, the osmotic minipumps were connected to the i.c.v. cannula tubing and were subcutaneously implanted on the rat’s back. For post-surgery pain management, subcutaneous injections of long-acting Buprenorphine at a dosage of 0.65 mg/kg were administered, with a second injection of the same dosage given 72 h later. Following a 10-day recovery period, behavioral testing was performed.

### Cloning

Rat Tacr3-mcherry (NM_017053.1) was sub-cloned from a synthesized template (VectorBuilder), and the whole construct was amplified by PCR using the primers: GCTCTAGAGCCACCATGGCCTCAGTCC, AACATGCATGCTTACTTGTACAGCTCGTCC. The resulting construct was cloned into the Sindbis vector pSinRep5 between XbaI and PaeI restriction sites. The rat Tacr3-IRES-EGFP construct was first sub-cloned into pHA-IRES-EGFP between BcuI and PstI, and TacR3 was PCR amplified from a template using the primers: GACTAGTGCCACCATGGCCTCAGTCC, GCACTGCAGTTAGGAATATTCATCCACAGAGGTA. The whole TacR3-IRES-EGFP construct was then amplified using specific primers (GCTCTAGAGCCACCATGGCCTCAGTCC, AACATGCATGCTTACTTGTACAGCTCGTCC) and cloned into the pSinRep5 Sindbis vector between at the XbaI and PaeI restriction sites. After successful ligation, the plasmids were linearized for transcription, and recombinant RNA transcripts were then synthesized using the SP6 promoter and transfected into BHK cells (Supplementary Fig. 2).

### Virus preparation and neuron infection

Sindbis virus was prepared as described previously [27–29]. Briefly, plasmids containing the protein of interest (pSinRep5) and the helper plasmid (pDHtRNA) were linearized and purified using phenol-chloroform extraction, followed by ethanol precipitation. In vitro RNA transcription was performed using the mMESSAGE mMACHINE® SP6 Transcription Kit (Thermo Scientific, AM1340), and the RNA obtained was then purified using phenol-chloroform extraction, followed by isopropanol precipitation.

For each nucleofection, a total of 10 × 10^6^ BHK-21 cells were electroporated and resuspended in 100 µl of Cell Line Nucleofector® solution (Lonza, VCA-1005), along with 10 µg of the transcript of interest and 10 µg of the helper RNA. Electroporation was carried out using the protocol for the BHK-21 cell line with the Amaxa Nucleofector II system. Immediately after electroporation, the cells were plated onto a 150 mm dish and maintained at 37 °C in 5% CO_2_. BHK 21 (Clone 13) from Hamster Syrian kidney was from Sigma-Aldrich (#85011433). This commercial cell line is tested by the ECACC for mycoplasma.

At 48–72 h post nucleofection, the medium containing viral particles was recovered and concentrated by ultracentrifugation for 2 h on a 20% sucrose cushion at 25,000 rpm using a SW28 rotor. The supernatant was discarded, and the pellet was resuspended in 5% Fetal Bovine Serum (FBS) in a neurobasal medium (NBM: Life Technologies, 21103049). The virus was then stored at −80 °C for further use.

### FORTIS: fluorescence monitoring with a microplate reader

Neuronal cultures in 96-well plates maintained at 37 °C in 5% CO_2_ and at a controlled humidity were transferred to a SPARK Multimode Microplate reader (Tecan), and after obtaining a baseline recording of 5–60 min, the cultures were treated as desired. For osanetant treatment, primary neuronal cultures (15 DIV) were infected over 24 h with Sindbis virus expressing SEP-GluA1 (a pH-sensitive fluorescence protein). The next day, the media was replaced with an equilibrated bathing solution, and the cells were incubated for 10 min before reading in the SPARK reader for 30 min. A portion of the plate was then treated with osanetant (final concentration 100 nM), and any fluorescence changes were recorded every 30 min for 4 h. For the induction of chemical LTP (cLTP), glycine was added for 5 min at a final concentration of 200 µM, while control samples were treated with the bathing solution without glycine. After a 5-min incubation, the solution was replaced with the bathing solution (glycine-free), and the plate was read every 30 min over 4 h. Fluorescence readings were obtained using 475Ex/535Em nm filters.

### Multielectrode array

The electrophysiological activity was recorded using an Axion Maestero Edge recording system with 16 extracellular recording electrodes and a ground electrode per well on a 24-well multielectrode array (MEA: Axion Biosystems, M384-tMEA-24W). Neurons were plated at a density of 30,000 per well in NBM (5 μL) with 10% FBS (Atlanta Biologicals, S11550), and they were allowed to attach to the plate for 2 h, after which 300 μL of serum-free NBM was added. At 9 DIV, 50% of the medium was changed and supplemented with BrainPhys medium, and at 12 DIV, 50% of the medium was changed with supplemented NBM. From 13 DIV, neurons were recorded using Axion AxIS Navigator software over 10–120-min intervals. Electrical activity was measured with an interface board at 12.5 kHz, digitized, and transmitted to an external computer for data acquisition and analysis.

All voltage data were filtered using dual 200 Hz (high pass) and 3000 Hz (low pass) filters, and action potential thresholds were set automatically using an adaptive threshold for each electrode (>6 standard deviations from the electrode’s mean signal). Waveforms collected with the Axion AxIS Navigator were exported to a Plexon Offline Sorter (v4) for automatic completion, and the Principal Components Analysis (PCA) was plotted on the waveforms for each electrode. The K-means clustering algorithm was used to split the waveforms by source units, allowing per-neuron analyses using NeuroExplorer (v5), Axion Neural Metric Tool, and ad-hoc Python scripts.

A *cross-correlation analysis* was used to assess the connectivity between pairs of neurons [30], with correlograms presenting the conditional probability of a spike from one neuron, given that a spike occurred in a reference neuron. Only sorted neurons with firing rates above 20 spikes per recording (1/30 Hz) were filtered for cross-correlation calculations. Cross-correlograms were calculated between each possible pair of neurons in the same well using NeuroExplorer software, and the average cross-correlogram for each treatment was calculated considering all the neuron pairs under the same treatment for each record separately. The average cross-correlogram peak for each treatment and recording was also calculated. All data analysis was performed using ad-hoc in-house Python scripts.

### RT-PCR

RNA was extracted from the neuronal cell lysate using the NucleoSpin RNA mini kit (Macherey-Nagel), and this RNA was reverse transcribed with All-In-One 5X RT MasterMix (ABM, #G592), diluting the resulting cDNA to 100 ng/µl. Gene-specific primers were designed with the Primer-BLAST NCBI tool, and their sequences are listed. Real-time PCR (RT-PCR) was carried out on a LightCycler® 480 (Roche) and using SYBR Green PCR Master Mix (Applied Biosystems), with an initial denaturation at 95 °C for 20 s, followed by 40 cycles at 95 °C for 3 s and 60 °C for 30 s. Each sample was run in triplicate, and the 2-∆∆Ct method [31] was used for the relative quantification of gene expression, with changes in gene expression normalized to an internal control gene (Actin). The following primers were used: TACR3 Forward CACAAGCGCATGAGAACTGT; TACR3 Reverse: AAGTTCTGGAAGCGGCAGTA; Actin Forward: CCCTACAGTGCTGTGGGTTT; Actin Reverse: GCAAGGAGTGCAAGAACACA.

### Chemical LTP (cLTP)

A protocol described previously was followed for cLTP induction with minor changes [29, 32]. Specifically, neuronal cultures at 37 °C and in 5% CO_2_ were incubated in 200 μM glycine-containing extracellular solution at pH 7.4 (in mM): 129 NaCl, 4 KCl, 4 CaCl_2_, 10 HEPES, 10 Glucose. The controls were incubated in a glycine-free extracellular solution alone (vehicle).

### Dendritic spine analysis

(i) *Primary neurons*: To assess the overall morphology of dissociated neurons, cells (20–24 DIV) were infected with the EGFP Sindbis virus for 24 h to visualize the dendrites and dendritic spines. The cells were fixed in fresh 4% PFA in PBS for 10 min at room temperature and washed three times with PBS. The cells were then covered with Prolong Gold Antifade Reagent (Thermo Fisher Scientific, P36934), and after 24 h, they were visualized on a Zeiss LSM880 Airyscan confocal microscope equipped with an Argon 488 nm laser line. A tile-scan application was used to obtain images of whole neurons (10x) or dendrites (63x), quantifying spines using Imaris 9.7.2 software (Bitplane Inc.) and dividing the number of spines by the corresponding dendritic length to calculate the spine density on each dendrite.
  To test the effect of testosterone and osanetant on dendritic spines, primary hippocampal cultures from rats were infected with a Sindbis virus expressing EGFP at 16 DIV. At 20 h post-infection (hpi), the neurons were exposed to either osanetant (100 nM) or testosterone (10 nM) for 2 h in a growth medium. In rescue experiments, neurons were initially exposed to testosterone (10 nM) for 2 h, and then osanetant (100 nM) was added for a further 2-h incubation. The neurons were then fixed in 4% PFA and washed in PBS with 4% sucrose for 10 min and then three times with PBS prior to mounting the coverslips with ProLong Gold™ Antifade Mounting medium (Invitrogen™) and visualizing them on an Olympus IXplore SpinSR10 microscope.
(ii) *Brain slices. Intracellular injections of Lucifer Yellow*: Rats were anesthetized with pentobarbital (0.04 mg/kg) and transcardially perfused with 300 ml of 4% PFA (pH 7.4) prior to removing their brain. Each brain was coded (codes were not broken until after the quantitative analysis), post-fixed in 4% PFA (pH 7.4) for 24 h, and coronal microtome sections (150 µm: Leica VT1000 S Vibrating blade) were labeled with 4,6-diamidino-2-phenylindole (DAPI: Sigma D9542) for 1–2 min. Cells in the ventral dentate gyrus (DG) and the lateral nucleus of the amygdala were injected individually with 4% Lucifer Yellow (CH: Sigma-Aldrich) in 1 M LiCl (pH 7.4) by passing a steady hyperpolarizing current through the electrode (−0.5 to −1.0 nA).

#### Tissue processing

Following injection, the sections were probed overnight with an antibody specifically targeting Lucifer Yellow (1:200 Rabbit: Thermo Fisher Scientific #A-5750) and then for 4 h with an Alexa Fluor Plus 488 conjugated Goat anti-Rabbit IgG (H + L) secondary antibody (1:1000: Invitrogen, # A32731). The sections were preserved and then mounted in fresh ProLong Gold antifade reagent (Invitrogen, Eugene, OR), the slides were left in the dark at room temperature for 24 h to cure the mounting medium, and finally, the coverslips were sealed using nail polish.

#### Confocal microscopy

Sections were visualized on a Zeiss laser scanning multispectral confocal microscope equipped with an argon laser. The acquired image stacks had a physical size of 76.9 × 76.9 μm and a logical size of 1024 × 1024 pixels. These stacks consisted of 100–350 image planes captured through a 63× glycerol immersion lens (NA 1.3, working distance 280 μm, and refraction index 1.45). To optimize the imaging, a zoom factor of 3.2 was calculated, resulting in a voxel size of 75.1 × 75.1 × 136.4 nm with a z-step of 0.14 μm. For each rat (5 neurons per rat), 1–5 randomly selected dendrites were scanned from the soma to the tip. Subsequently, the acquired stacks were processed using a 3D blind deconvolution algorithm (ClearView™ GPU Accelerated Deconvolution), applying 10 iterations to reduce the impact of out-of-focus light.

#### Spine density

Dendrites were traced using the Neurolucida 360 software (MicroBrightField Inc., Williston, VT). Dendritic spine densities were determined on granular neurons in the DG or on pyramidal-like neurons in the lateral nucleus of the amygdala, traced from their proximal to distal tips and marking the presence of spines during the tracing process. This analysis was performed on five neurons per rat for each area, and all protrusions observed were considered spines and included in the analysis without applying any factors to correct the spine counts. The reconstructed data were then exported to Neurolucida Explorer (MicroBrightField Inc., Williston, VT) for further quantification. The spine density was automatically calculated, as indicated previously.

#### Spine morphology

Spine head volume was measured using Imaris 9.7.2 software (Bitplane AG, Zurich, Switzerland) [33, 34] and for each dendritic segment, various intensity thresholds were applied to generate a data model that was visualized as a solid surface using the IsoSurface module. Subsequently, the solid surface corresponding to the contour of each spine head was selected. The three-dimensional image of each dendrite was rotated and carefully examined to verify the accuracy of the solid surface selected for each spine head. Spines with no visible head were extremely rare and were not included in the analysis.

### Electrophysiology

#### In vivo electrophysiology

For the in vivo studies, rats were anesthetized with urethane (1.6 g/kg i.p.) to assess LTP induction in the DG. Surgical procedures and recordings were performed while the animals were situated in a Kopf stereotaxic device. Field potentials were obtained using Nichrome microelectrodes (<1 MΩ, 120 μm thick), and the perforant pathway was stimulated using a bipolar electrode (World Precision Instruments) at double the threshold intensity to elicit a response (10–50 μA). The experimental protocol consisted of a 10-min baseline period to establish stable activity, with the stimulation pathway activated at 0.5 Hz. Subsequently, three stimulation trains of 100 Hz were delivered for 500 ms each, with a 2 s interval between trains to induce LTP. Following LTP induction, the pathway was stimulated again at 0.5 Hz for 30 min, and the average evoked field potential was calculated every minute (30 stimuli). The slope of the evoked field potential was measured and plotted, with the mean slope during the control period considered as 100%.

#### Slice electrophysiology

Rats were anesthetized with sodium pentothal (20 mg/kg of body weight, intraperitoneal), decapitated, and their brain was rapidly removed and placed in oxygenated, ice-cold dissection solution: 10 mM D-glucose, 4 mM KCl, 26 mM NaHCO_3_, 233.7 mM sucrose, 5 mM MgCl_2_, 1:1000 Phenol Red. Coronal microtome slices (300 μm, Leica VT1000 S Vibrating blade) were placed in a recovery chamber containing Artificial CSF (aCSF) at 24–26 °C for at least 1.5 h before recording. The aCSF, with an osmolarity adjusted to 290 mOsm, was used for recovery and recording: 119 mM NaCl, 2.5 mM KCl, 1 mM NaH_2_PO_4_, 11 mM glucose, 1.2 mM MgCl_2_, 2.5 mM CaCl_2_. A concentric bipolar platinum-iridium stimulation electrode and a low-resistance glass recording microelectrode filled with aCSF (3–4 MΩ resistance) were placed in the middle molecular layer to record extracellular field excitatory postsynaptic potentials (fEPSPs). In each slice, an input-output (I/O) curve was recorded to compare the basal synaptic transmission in different animals.

### Antibodies

The primary antibodies used here were raised against*:* NK3R (TACR3, Assay Biotech R12-3093), NeuN (polyclonal: Synaptic Systems, 266 006), GluA1 (Cell signaling 13185 S or Abcam #ab31232), phospho-CaMKII, T286 (Millipore #05-533), CaMKII (Sigma Aldrich #C6974), PSD95 (NeuroMab 75-028), synaptophysin, (Millipore #MAB329), β-actin (Cell signaling 4970 S or Cell Signaling Technology 4970 S), Phospho-(Ser) PKC Substrate Antibody (Cell signaling #2261) GAPDH (Santa Cruz Biotechnology sc-47724). The secondary antibodies used were: anti-mouse and anti-rabbit IgG HRP-linked secondary antibodies (Cell Signaling 7076 S and 7074 S), goat anti-Chicken Alexa Fluor® 633 (Invitrogen 10444562), cross-adsorbed Alexa Fluor 594 and 488 goat anti-Mouse IgG (H + L) antibodies (Thermo Scientific A-11005 and A-21121), and cross-adsorbed Alexa Fluor 488 and 594 goat anti-Rabbit IgG (H + L) secondary antibodies (Thermo Fisher Scientific A-11008 and A-11012).

### Drugs

The drugs used in this study were: glycine (Bio Lab Ltd UN #071323), strychnine (Sigma-Aldrich #S0532-5G), senktide (Tocris #1068), osanetant (Sigma-Aldrich SML0798-25MG), and testosterone propionate (Sigma-Aldrich # 86541-5 G).

### Statistical analysis

Analyses were carried out using GraphPad Prism software (version 8.00, GraphPad Software, La Jolla, CA, USA). A Kolmogorov-Smirnov normality test was used to assess the distribution of the datasets, applying parametric or non-parametric analysis as appropriate. The data was presented as the mean ± standard error of the mean (SEM), and the number of animals, cells, spines, or cultures are indicated in each figure. All experiments were carried out at least three times and the data presented are the combined results of all these repetitions. The statistical tests used and the *P* values are indicated in the figures or their corresponding legends.

We did not assume equal variances among groups, and as a precautionary measure, non-parametric tests were used for statistical comparisons unless explicitly stated otherwise. All tests are two-sided. Adjustments for multiple comparisons were made using the Bonferroni method. Throughout the study, ‘center values’ are defined as the mean, and error bars represent the standard error of the mean (SEM).

#### Sample size and exclusion criteria

No a priori sample size calculation was performed to detect a pre-specified effect size for this study. The sample size was determined based on previous similar experiments and the feasibility of the study within the given time frame. Additionally, no animals were excluded from the analysis; all animals that were subjected to experimental conditions were included in the final data set. The inclusion of all subjects and the absence of a pre-calculated sample size should be considered when interpreting the study results.

The other methods used in the study are described in the Supplemental Materials*and Methods*.

## Results

To investigate the molecular mechanisms that potentially underlie anxiety, we assessed the anxiety-like behavior of male Wistar rats (3 months old) in the EPM [35, 36] (Fig. 1a), a paradigm in which high levels of anxiety are reflected by avoidance of the open arms of the maze and preferential exploration of the closed arms [37]. Substantial variability was evident among the rats in the time spent in the open or closed arms during the trial (Fig. 1b). Accordingly, the ventral hippocampus was extracted from SA and MA rats undisturbed for two weeks, and their transcriptional profiles were analyzed by microarray RNA expression analysis (Fig. 1c–g and Supplementary Fig. 1). The ventral hippocampus was examined since it is crucial in regulating anxiety-related behaviors [38, 39], and its dysregulation has been implicated in the modulation of anxiety-like behaviors and the development of anxiety disorders [36, 39–42].

### Rats with severe anxiety-like behavior exhibit hippocampal TACR3 deficiency

A differential expression analysis (with an absolute log-fold change >1 and a *p*-value < 0.05) revealed 78 up-regulated and 94 down-regulated genes when SA and MA rats were compared (see Fig. 1c, d). To further understand the biological functions of these DEGs, we conducted KEGG and GO enrichment studies. KEGG pathways connected to synapses and synaptic plasticity were notably enriched (see Fig. 1e), while the enriched GO biological processes included neuropeptide signaling, modulation of synaptic transmission, and the tachykinin receptor signaling pathway. Additionally, GO cellular component terms that were enriched implicated glutamatergic synapses, dendrites, and dendrite membranes. These findings highlight and support the notion that severe anxiety influences genes involved in synaptic activity.

Remarkably, the Neuroactive ligand-receptor interaction pathway (KEGG pathway rno04080) emerged as the most significant pathway in our KEGG analysis (see Fig. 1e). Within this pathway, we identified several proteins associated with the modulation of anxiety-related behaviors, such as Cortistatin [43], Neuronal Nicotinic Acetylcholine Receptor [44], Opioid-Related Nociceptin Receptor 1 [45], Tachykinin Receptor 3 [21], Somatostatin receptor 1 [46], Tachykinin Precursor 1 [47] and Neurotensin [48]. This finding suggests that altering the expression of genes involved in neuroactive ligand-receptor interactions may contribute to the development of an SA-like phenotype in rats. Interestingly, the TACR3 gene was prominent within this KEGG pathway, showing a marked downregulation in SA rats (see Fig. 1c). TACR3 is a membrane receptor for the tachykinin neuropeptide neuromedin-K (also known as neurokinin B), and it is involved in 5 GO biological processes. In addition, it is associated with 2 GO enriched cellular components: the neuronal cell body membrane and the dendrite membrane (see Fig. 1f, g). Another gene that was associated with several enriched KEGG and GO terms is CAMK2B, which encodes Calcium/Calmodulin Dependent Protein Kinase II Beta (more commonly known as CaMKII) and interestingly, CAMK2B expression was elevated in SA rats (Fig. 1c). Significantly, this protein was involved in the most prominently altered KEGG pathways, in particular those strongly linked to synaptic function. Moreover, CAMK2B is part of one enriched GO biological process and three enriched GO cellular components (Fig. 1e–g).

### Hippocampal TACR3 is modulated by sex hormones

**Fig. 2:**
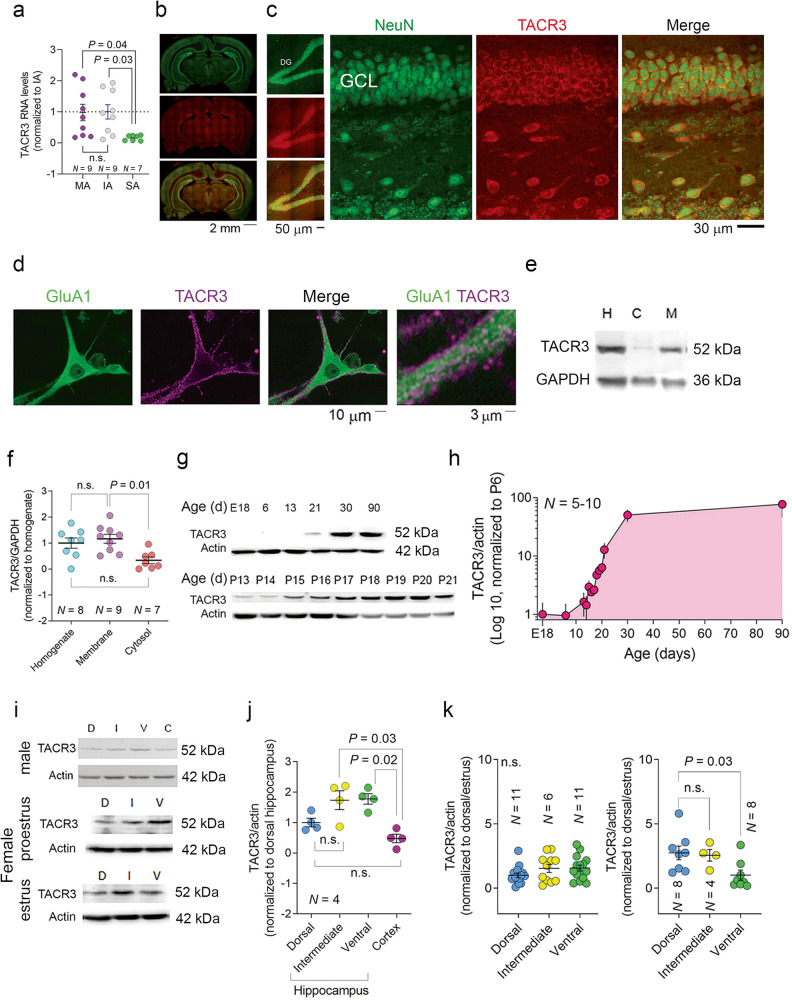
TACR3 expression depends on sex hormones. **a** TACR3 expression in the ventral hippocampus analyzed by qPCR. Total RNA was isolated from the ventral hippocampus of male SA, IA, and MA rats and analyzed by qPCR using TACR3-specific primers. The relative TACR3 mRNA expression was calculated using the 2^-ΔΔCt method and presented as dots for individual values and as the mean ± SEM. Statistical significance was determined using the Kruskal-Wallis test, followed by Dunn’s multiple comparisons tests. N represents the number of rats. **b** Representative images of TACR3 immunohistochemistry in coronal brain sections of adult male rats. TACR3 expression is shown in red (Alexa 594), and neurons (green, Alexa 488) were identified with NeuN. **c** *Left*: Confocal images of the dentate gyrus. *Right*: High magnification (60x) images demonstrating TACR3 expression in red (Alexa 594) and labeling for a neuronal marker (NeuN) in green (Alexa 488). **d** Confocal image of TACR3 labeling in primary hippocampal neurons. Neurons were stained with a TACR3-specific primary antibody that was detected with an Alexa594 fluorescently labeled secondary antibody. TACR3 staining (purple) was observed in the cell body and around the dendrites of hippocampal neurons, with a punctate distribution. Co-staining with GluA1 (green, Alexa488) does not indicate co-localization. **e** Representative Western blot of TACR3 expression of hippocampal samples fractionated into cytosolic and membrane fractions. **f** Quantification of TACR3 protein expression relative to GAPDH expression in the homogenate (H), cytosol (C), and membrane (M) fractions. Each dot represents the values for a single rat and the data are also presented as the mean ± SEM. Statistical significance was determined using a Kruskal-Wallis test followed by Dunn’s multiple comparisons tests, and *N* represents the number of rats in each group. **g** Western blot of TACR3 protein expression in the hippocampus during development. Hippocampal tissue lysates were collected from male rats at different stages of development. **h** The TACR3 protein expression of normalized to β-actin across different developmental stages. The results show a gradual increase in TACR3 expression during development, representing the data as the mean ± SEM. *N* is the number of rats per age (*N* = 5 for E18, P6, P30, P90; *N* = 10 for the rest of the time points). **i** Representative Western blots of TACR3 expression in cortex and hippocampus lysates from adult male rats, and female rats at the estrous cycle’s proestrus and estrus stages. **j** Analysis of TACR3 expression in the hippocampus and cortex. TACR3 protein expression was analyzed in the dorsal hippocampus, ventral hippocampus, the intermediate area between them, and in the cortex of adult male rats. Each dot represents the value of a single rat, and the data are also presented as the mean ± SEM. Statistical significance was determined using the Kruskal-Wallis test, followed by Dunn’s multiple comparisons tests, and *N* represents the number of rats in each group. **k** The figure shows TACR3 expression in the hippocampus of female rats at different stages of the estrous cycle, with proestrus on the left and estrus on the right. Each dot represents the value of a single rat, and the data are also presented as the mean ± SEM. Statistical significance was determined using the Kruskal-Wallis test, followed by Dunn’s multiple comparisons tests, and *N* represents the number of rats in each group.

RT-PCR analysis confirmed a significant reduction in TACR3 expression in SA rats, approximately 5-6-fold relative to IA and MA rats (Fig. 2a). Through immunofluorescence, TACR3 was seen to be expressed throughout the rat brain, with particularly prominent expression in the hippocampal formation (Fig. 2b, c). We further validated the predominant localization of TACR3 to the membrane through immunofluorescence of primary neurons and the analysis of cell fractions in western blots (Fig. 2d–f). Considering the critical role of TACR3 in CHH, where patients experience lower testosterone levels [16–18] and anxiety [49], we studied the expression of TACR3 during sexual maturation. When TACR3 levels were analyzed in the hippocampus of rats throughout development (Fig. 2g, h), a remarkable upregulation of hippocampal TACR3 expression was detected during adolescence, with a 50-fold increase at 30 days of age. In adult 3-month-old males, TACR3 expression was more pronounced in the ventral hippocampus and in the transitional region between the ventral and dorsal hippocampus relative to the dorsal hippocampus (Fig. 2i, j). Notably, this differential expression was not evident in 3-month-old female rats during estrus, and it was inverted during the proestrus stage (Fig. 2i–k), with weaker expression in the ventral hippocampus compared to the dorsal and intermediate regions. These findings suggest that TACR3 expression in the hippocampus is dynamic and influenced by sex hormones.

**Fig. 3:**
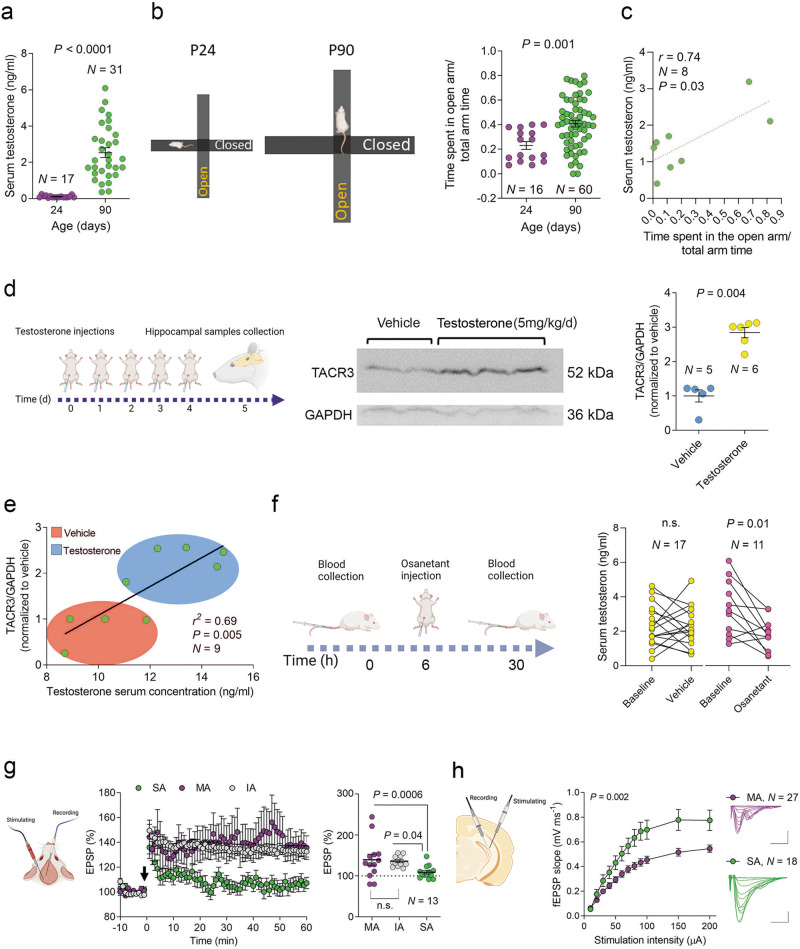
The interaction between TACR3 expression, testosterone levels, and rat anxiety-like behavior. **a** Using ELISA, Serum testosterone levels were measured in male rats on postnatal days 24 (P24) and 90 (P90). The graph displays the values for each rat and the mean ± SEM testosterone levels (ng/ml) in each age group. The testosterone levels increased significantly from P24 to P90, and the statistical significance was determined using the Mann-Whitney test. *N* represents the number of rats in each group. **b** *Left*: A schematic representation of the elevated plus-mazes used for behavioral testing of P24 (left) and P90 (right) male rats. The maze size was proportionally similar to the size of the rats, ensuring appropriate scaling for the experimental conditions. *Right*: The graph illustrates the scores from the EPM test in P24 and P90 rats. P90 rats exhibited a significantly higher score, spending more time in the open arms of the maze compared to P24 rats. This observation suggests P90 rats display less intense anxiety-like behavior as they have a greater propensity to explore the open areas of the maze. Each dot represents the score of a single rat in the test, and the data are also presented as the mean ± SEM. The statistical significance was determined using the Mann-Whitney test, and *N* represents the number of rats in each group. **c** Correlation between the serum testosterone levels and EPM score for individual male rats (3 months old). Statistical significance was determined using Pearson’s correlation, and *N* represents the number of rats. **d** *Left*: Scheme of the experimental design. Male rats were randomly assigned to a testosterone or control group (vehicle), with the rats in the testosterone group receiving daily subcutaneous injections of testosterone propionate (5 mg/kg) over five consecutive days. On the sixth day, the rats were sacrificed, and the hippocampus was collected for analysis. *Middle*: Western blot of TACR3 expression in the hippocampus. *Right*. A graph showing the quantification of TACR3 protein expression demonstrating a significant upregulation of TACR3 expression in the hippocampus of testosterone-treated rats relative to the control rats. Each dot represents the value of one rat and the statistical significance was determined using a Mann-Whitney test. *N* represents the number of rats in each group. **e** The correlation between serum testosterone levels and hippocampal TACR3 expression for individual male rats (3 months old) demonstrating a positive correlation between serum testosterone and hippocampal TACR3 expression. Statistical significance was determined using Pearson’s correlation and *N* represents the number of rats. **f** *Left*: Scheme of the experimental design used to examine the effect of osanetant on the serum testosterone levels in rats. Rats were randomly divided into two groups: a treatment group receiving a single intraperitoneal dose of osanetant (5 mg/kg) and a control group receiving the vehicle alone. Blood samples were collected from each rat via tail puncture 6 h before and 24 h after the treatment, measuring the testosterone levels in the serum. The graph on the right displays the individual values of the rats before and after osanetant or vehicle treatment. Statistical significance was determined using a Paired *t* test, evaluating the changes within each group, and *N* represents the number of rats. **g** *Left*: Representation of a rat head indicating the location of the stimulating and recording electrodes. *Middle*: In vivo LTP in the dentate gyrus of rats categorized as MA, IA, and SA, highlighting the LTP impairment in SA rats. *Right*: Quantification of the EPSP changes in the last 10 min of the recording. Each dot represents the value of a single rat and *N* represents the number of rats. Statistical significance was determined using a Kruskal-Wallis test followed by Dunn’s multiple comparisons tests. **h** *Left*: A diagram illustrating a hippocampal slice, delineating the positioning of both the stimulating and recording electrodes during field potential recording within the dentate gyrus. *Middle*: Input-output curves representing field excitatory postsynaptic potentials (fEPSPs) induced by stimulation of perforant path axons in slices of MA and SA rats. *Right*: Overlay of sample fEPSPs at increasing stimulation intensities from 10 to 200 μA. *P*-values were calculated using a two-way ANOVA, and N represents the number of slices. The scale bars applicable to all panels are set at 0.5 mV and 20 ms, and the data are presented as the mean ± standard error of the mean (SEM), as shown by the error bars.

We hypothesized that rats with lower TACR3 expression in the hippocampus and lower serum testosterone levels would display heightened anxiety-like behavior in the EPM. Our hypothesis was confirmed through several findings. Firstly, we observed significantly lower serum testosterone in young rats (24 days) than in adult rats (90 days: Fig. 3a), as well as an increase in anxiety-like behavior in the EPM adjusted for size [50] (Fig. 3b). In addition, we identified a significant linear correlation between serum testosterone levels and anxiety-like behavior in adult rats (Fig. 3c). To further establish a link between TACR3 and testosterone, testosterone was administered (intraperitoneally) to adult male rats and it induced stronger TACR3 expression in the ventral hippocampus (Fig. 3d). A linear correlation was evident between serum testosterone levels (either endogenous or induced by injection) and TACR3 expression in the hippocampus (Fig. 3e). Notably, administration of the TACR3 inhibitor osanetant (5 mg/kg) significantly reduced serum testosterone levels within 24 h in adult rats (Fig. 3f). These outcomes suggest that testosterone and TACR3 are related reciprocally, whereby they mutually influence each other to modulate anxiety-like behavior. Upon intracerebroventricular administration of osanetant to 3-month-old male rats via mini osmotic pumps for a 10-day period, we observed no significant changes in anxiety-related behaviors (Supplementary Fig. 3a). This observation suggests that the influence of TACR3 on anxiety-related behaviors may either be more significant during the earlier stages of brain development, or it may necessitate longer periods of inactivity to manifest changes in anxiety-related behavior.

### TACR3 activity determines the expression of LTP in vivo and in vitro

Orchidectomized males have less testosterone, resulting in deficient LTP [51], and the addition of testosterone to slices from orchidectomized males in vitro notably amplified the excitatory postsynaptic potentials, mirroring the changes seen during LTP [52]. Consequently, SA rats that are characterized by diminished serum testosterone might exhibit compromised LTP. To test this assumption, we obtained recordings in vivo from rats that display a spectrum of anxiety-like behaviors, inserting stimulating electrodes into the perforant pathway and recording from the DG, inducing LTP by delivering three high-frequency (100 Hz) stimulation trains. We focused on the DG because this region is implicated in the pathophysiology of anxiety disorders [53, 54]. LTP was apparent in the brains of the less anxious rats (both MA and IA), but it was missing in SA rats (Fig. 3g). In addition, the mean synaptic responses of SA rats remained on par with their pre-LTP induction baseline levels. When basal synaptic transmission was assessed in the DG of acute slices from SA and MA rats, elevated I/O curves were obtained from the slices from SA rats (Fig. 3h), suggesting an innate enhancement in synaptic transmission that might make LTP induction more challenging [55].

### Reduced TACR3 activity leads to CaMKII activation associated with PKC signaling

**Fig. 4:**
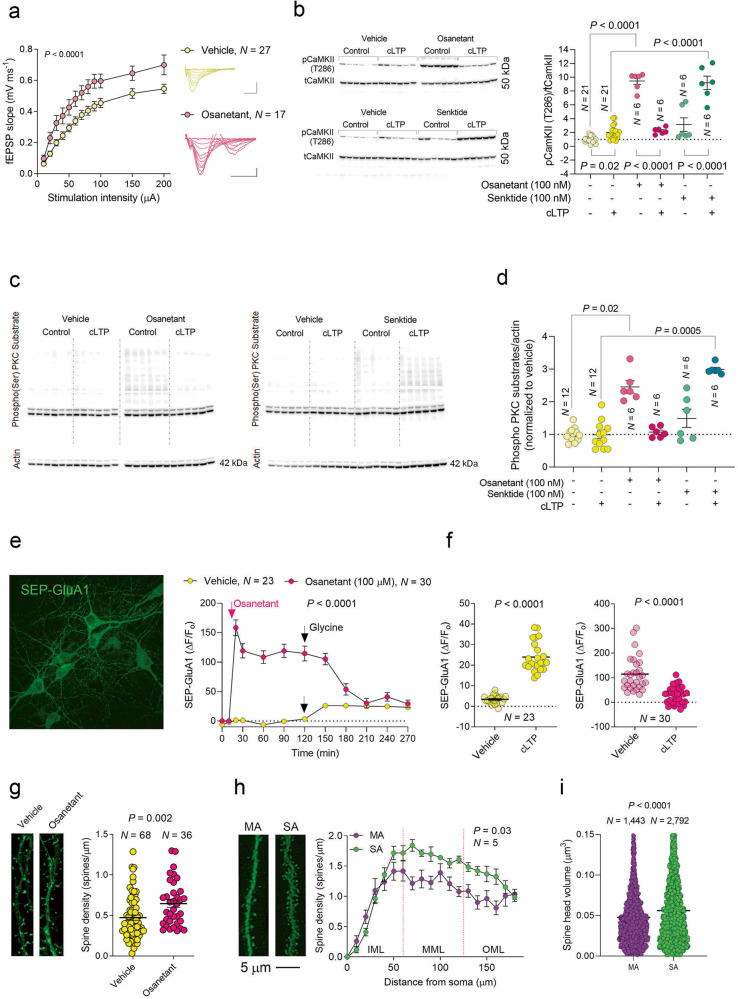
Impact of TACR3 deficiency or inhibition on synaptic connectivity and LTP. **a** *Left*: Input-output curves representing field excitatory postsynaptic potentials (fEPSPs) induced by stimulation of the perforant path axons in slices treated with osanetant or vehicle. *Right*: Overlay of sample fEPSPs at increasing stimulation intensities from 10 to 200 μA. The *P*-values were determined using a two-way ANOVA and N represents the number of slices. The scale bars applicable to all panels are set at 0.5 mV and 20 ms, and the data are presented as the mean ± standard error of the mean (SEM), as shown by the error bars. **b** *Left*: Western blots probed for phospho CaMKII (T286) following treatment with osanetant and senktide. *Right*: Phospho CaMKII/total CaMKII levels after different treatments. Each dot in the graph represents the value of a single culture and the statistical significance was determined with a one-way ANOVA followed by Tukey’s multiple comparisons tests. The difference between senktide and the vehicle is not statistically meaningful, with *N* representing the number of cultures in each group. **c** Western blot analyzing the phospho PKC substrates in cultures treated with osanetant or senktide before cLTP induction. **d** Quantification of the phospho PKC substrates. Each dot represents a single culture, and the statistical significance was determined by one way ANOVA followed by Tukey’s multiple comparisons tests. *N* indicates the number of cultures in each group. **e** *Left*: Primary hippocampal neurons expressing SEP-GluA1, a fluorescent marker for that AMPAR subunit at the neuronal surface. *Right*: Quantification of changes in surface SEP-GluA1 following osanetant treatment and cLTP induction. *N* represents the number of cultures. **f** Changes in surface AMPAR expression in neurons pretreated with the vehicle (left, yellow) or osanetant (right, pink), following cLTP induction. Each dot in the graph represents the value of a single culture and *N* represents the number of cultures. Statistical significance was determined using a paired *t* test. **g** *Left*: High-magnification (63x) maximum projection confocal image of dendrites and spines. *Right*: Spine density after treatment with vehicle or osanetant. *N* represents the number of neurons, and the statistical significance was determined using a Mann-Whitney test. **h** *Left*: Dendrites of granular neurons injected with Lucifer Yellow. *Right*: Sholl analysis demonstrating the relationship between spine density and the distance from the soma. *N* represents the number of rats in each group and the statistical significance was determined by two-way ANOVA. **i** Spine head volume in granular neurons of MA and SA rats. *N* represents the number of spines, and the statistical significance was determined using a Mann-Whitney test.

To explore whether we can replicate the association between basal synaptic transmission and anxiety using drugs, we inhibited TACR3 with osanetant (100 nM) for 1 h before the recording and during the recording and analyzed the basal synaptic transmission in acute slices through field recordings in the DG. Osanetant enhanced the I/O curve from these slices (Fig. 4a), mirroring the patterns observed in SA rats and further substantiating the role of TACR3 in synaptic transmission. To shed light on the connection between TACR3 activity, synaptic transmission, and LTP, for one hour before triggering cLTP, we exposed primary neurons to either osanetant or senktide (a TACR3 antagonist and agonist, respectively, both at 100 nM). It should be noted that Tac3 RNA has been identified in rat hippocampal neurons at 14 DIV through high-throughput sequencing [56], indicating that TACR3 is likely to be activated to some extent in our experimental framework.

There is an established relationship between the rise in excitatory postsynaptic currents following cLTP induction and an increase in phosphorylated CaMKII (pCaMKII) [57]. Subtle molecular and cellular mechanisms linked to LTP have been effectively explored in cultured neurons using cLTP [29, 36, 58, 59]. This approach has advantages over conventional electrical LTP as it enables a larger number of synapses to be potentiated, simplifying the task of pinpointing the molecular and cellular changes associated with LTP. Postsynaptic CaMKII activity plays a pivotal role in potentiating synaptic transmission, and it is both necessary and sufficient to generate LTP [60]. In our experiments, cLTP induction produced a significant 2-fold elevation in CaMKII phosphorylation at T286 (Fig. 4b and Supplementary Fig. 5a). However, osanetant treatment led to a much more dramatic surge and a roughly 10-fold increase in pCaMKII (Fig. 4b).

Enhanced CaMKII activity was previously shown to trigger maximal LTP, thereby impeding further LTP [60]. In line with this, co-administration of osanetant and glycine (200 µM) [29, 61] did not provoke a strong upregulation of pCaMKII, as seen with osanetant alone, probably because the postsynaptic expression of active CaMKII mimics and blocks further LTP induction [60, 62, 63] (Fig. 4b). By contrast, neurons exposed to senktide did not display a significant upsurge in pCaMKII. However, combining cLTP induction with senktide treatment enhanced pCaMKII levels, emphasizing the critical role of TACR3 activity in promoting LTP (Fig. 4b). Complete agreement was noted between the changes in pCaMKII and phospho PKC substrates in neurons treated with either osanetant or senktide (Fig. 4c, d), supporting the PKC pathway mediating CaMKII phosphorylation as seen elsewhere [28].

The PKC pathway fulfills a crucial role in the trafficking of α-amino-3-hydroxy-5-methyl-4-isoxazolepropionic acid receptors (AMPARs) by phosphorylating S831 in the GluA1 subunit of these receptors [28, 64, 65]. CaMKII phosphorylates the same residue during LTP, significantly enhancing their synaptic expression [66]. We used FluOrescence Receptor TraffIcking Screening (FORTIS) to explore whether the TACR3 antagonist osanetant boosts the surface expression of AMPARs, an approach that enables the labeling, monitoring, and analysis of synaptic efficacy and plasticity in live neurons [29]. Surface AMPARs were selectively labeled with genetically encoded AMPAR reporters tagged with super ecliptic pHluorin (SEP-GluA1), and SEP-GluA1 fluorescence was monitored in real-time [28, 29, 67]. By normalizing the readings after exposure to the drugs to the basal readings (ΔF/F_0_), the ratios obtained reflect the increase (%) in AMPAR surface expression during the assay. A significant increase in the SEP-GluA1 signal was evident following osanetant treatment (Fig. 4e, f), yet when we induced cLTP, a reduction in the SEP-GluA1 signal was observed that suggested endocytosis of AMPARs (Fig. 4e, f). In the control cultures in which cLTP was induced without pre-treatment with osanetant, a typical increase in the SEP-GluA1 signal was seen (Fig. 4e, f) [29]. Overall, our results indicate that while TACR3 suppression boosts the surface expression of AMPARs, it obstructs additional LTP induction [29].

### The activity of TACR3 shapes neuronal connectivity and activity

An exciting observation emerged when we directly examined the effect of TACR3 activity on synaptic connectivity. Upon inhibiting TACR3 in primary hippocampal neurons by exposure to osanetant (100 nM, 24 h), a significant rise in spine density was noted in EGFP-expressing neurons (Fig. 4g), again suggesting that TACR3 inhibition could boost synaptic connectivity. Expanding these studies to SA rats that lacked TACR3, we measured spine density in the granular neurons of the ventral DG. Consistent with our findings in cultures treated with osanetant, a Sholl analysis revealed higher spine density in the middle and outer molecular layers of these rats (Fig. 4h). Furthermore, we observed a significant difference in the spine head volume in SA rats relative to MA rats (Fig. 4i). Interestingly, the microstructural changes observed did not align with any alteration to the DG volume (Supplementary Fig. 3c). Moreover no elevation in spine density was noted on the lateral amygdala neurons (Supplementary Fig. 3d), which were also studied given the amygdala’s involvement in anxiety [68]. Consequently, these findings might be specific to hippocampal circuits. These patterns suggest that inhibiting or lacking TACR3 may potentially enhance spine density by activating the PKC pathway, which is known to facilitate dendritic spine growth [65].

**Fig. 5:**
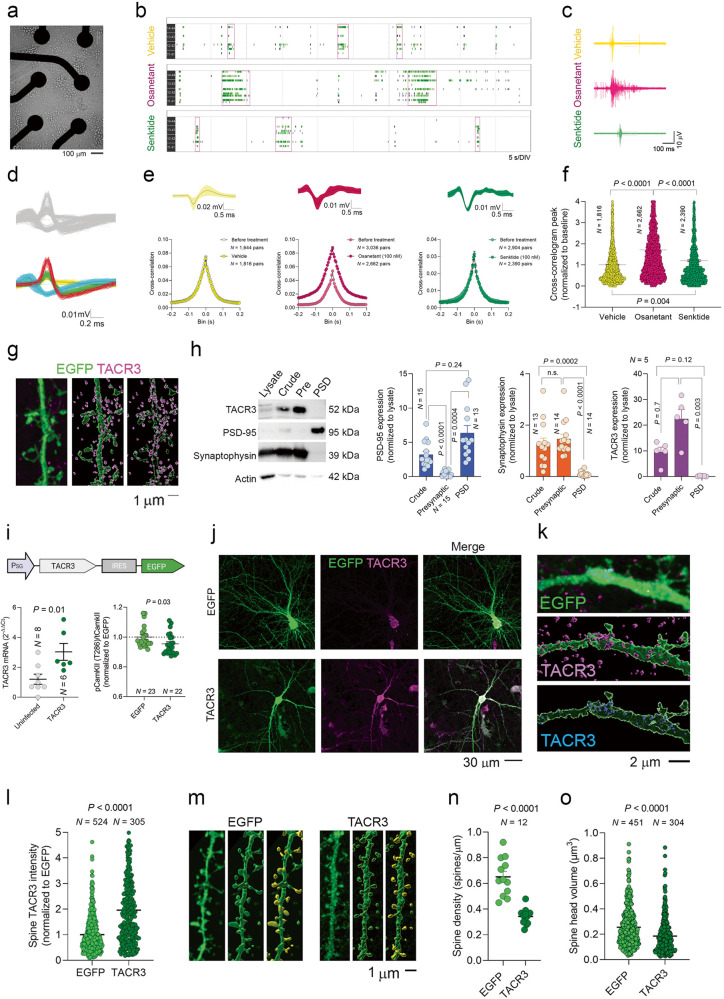
TACR3 function determines the cross-correlation among pairs of neurons. **a** Representative image of neurons cultured on a multielectrode array (MEA) acquired at 10x magnification on an inverted microscope. Each MEA consists of 16 planar electrodes arranged in a 4 × 4 grid. Neurons were cultured for 14 DIV, allowing a complex network of neurites and synapses to develop on the MEA surface. The MEA serves as a platform for non-invasive, long-term monitoring of neuronal activity. **b** Raster plots illustrating the firing activity of neurons on MEAs (DIV 14) after osanetant or senktide treatment: black lines represent individual electrode activity; green lines indicate the bursting activity of specific electrodes; and purple rectangles depict network bursts. Electrode numbers are displayed on the left side of the plots. **c** Examples of raw voltage traces obtained from single electrodes in MEA recordings using AxIS acquisition software. Gray bars indicate spike detection thresholds. **d** The upper panel displays the raw voltage traces recorded from a single electrode channel, whereas the lower panel demonstrates the same data after spike sorting using a principal component analysis (PCA). Before spike sorting, the raw voltage traces exhibit substantial noise and contain overlapping spikes from multiple neurons. Following spike sorting, individual spikes can be accurately differentiated and attributed to their respective neurons based on their waveforms. **e** The impact of osanetant and senktide treatment on the average cross-correlograms obtained from spike recordings that underwent spike sorting. Cross-correlograms were computed to assess the correlation between pairs of neurons based on their spike times. The upper panel displays an example of a single spike sorted throughout the recording duration. The lower panel presents the average cross-correlograms before treatment, representing the neuronal correlation in the absence of drug treatment and after treatment with osanetant or senktide, reflecting changes in neuronal correlation following drug administration. *N* represents the number of pairs of neurons. **f** The influence of different drugs on the peaks of cross-correlograms. Each data point on the graph represents the peak value of an individual cross-correlogram between two neurons. Statistical significance was determined using one-way ANOVA followed by Tukey’s multiple comparisons tests. **g** *Left*: Confocal projection image (63x) depicting a dendrite from a primary hippocampal neuron expressing EGFP (green) and immunostained with a TACR3 antibody (purple). *Middle*: Three-dimensional reconstruction of the dendrite and TACR3-positive punctae using the Surface module of Imaris software. *Right*: Dendritic contours illustrating the distribution of TACR3 labeled punctae around the dendrites rather than within them. **h** *Left*: Western blot analysis showing synaptic markers (PSD-95 and synaptophysin) in a synaptosomal preparation, along with TACR3 detected in the presynaptic compartment. *Right*: Quantification of the synaptic proteins, with each data point representing the values (normalized to crude synaptosome values) from the hippocampus of individual rats. Statistical significance was determined using one-way ANOVA followed by Tukey’s multiple comparisons tests. **i** *Top*: The construct for TACR3 overexpression containing TACR3 followed by an IRES and EGFP. *Bottom, left*: Quantifying TACR3 expression by qPCR in uninfected cultures compared to those infected with the TACR3 virus confirms successful overexpression. *Bottom, right*: normalized levels of pCaMKII/tCaMKII as determined by a microplate reader. Each data point represents the value of a single culture, and the statistical significance was determined using the Mann-Whitney test. *N* represents the number of cultures. **j** Representative examples of neurons expressing either EGFP alone or TACR3 along with EGFP, immunostained with a TACR3 antibody. **k** *Top*: Confocal projection image (63x) of a dendrite from a primary hippocampal neuron expressing TACR3 (TACR3-IRES-EGFP, green) and immunostained with a TACR3 antibody (purple). *Middle*: Three-dimensional reconstruction of the dendrite and TACR3-positive punctae using the Surface module of Imaris software, highlighting the presence of TACR3-positive puncta around the dendrite (purple). *Bottom*: Dendritic contours outlining the distribution of TACR3-positive punctae within the dendrites (blue). **l** Measurement of the TACR3 in dendritic spines of neurons expressing EGFP alone (EGFP) or neurons overexpressing both TACR3 and EGFP (TACR3). The statistical significance was determined using the Mann-Whitney test and *N* represents the number of spines analyzed. **m** *Left*: Dendrites from neurons expressing EGFP alone (EGFP) or neurons overexpressing both TACR3 and EGFP (TACR3). *Middle*: Three-dimensional structure of the same dendrites captured using the Surface module of Imaris software. *Right*: Dendritic spine heads were visualized using the Surface module of Imaris software, quantifying the spine density and spine head volume. **n**, **o** Quantification of spine density and spine head volume using Imaris software. Statistical significance was determined using the Mann-Whitney test and *N* represents the number of dendrites analyzed for spine density or the number of spines analyzed for spine head volume. Each data point represents the value obtained from a single dendrite or spine.

Building on our findings regarding the role of TACR3 in synaptic connectivity, we further investigated this using cross-correlation analysis. As such, primary neurons were grown on an MEA, the utility of which resides in their ability to monitor multiple neurons simultaneously, offering a more comprehensive understanding of the behaviors of intricate neural circuits (Fig. 5a–c) [69]. Cross-correlation analysis of distinguishable multi-unit activity is a technique to assess neuronal connectivity, the cross-correlogram providing insight into the temporal connections between individual neuronal activities [70, 71]. With this statistical instrument, we explored the effect of TACR3 manipulations on the connectivity among neurons within a network by analyzing data from thousands of individual neuronal pairs on the MEA electrodes. Neurons from 350 to 1500 μm apart were sorted using a PCA (Fig. 5d) [72]. Intriguingly, our analysis showed that osanetant markedly amplified the average cross-correlogram height (Fig. 5e, f), pointing towards an increase in the correlation between the activity of neurons within the network. Beyond its influence on cross-correlation, osanetant also produced intriguing shifts from the typical firing patterns of neurons, with a more significant fraction of spikes appearing within bursts (~40%) relative to neurons exposed to senktide or the vehicle alone (~20%: Supplementary Fig. 4a). Furthermore, the likelihood of burst events increased significantly upon TACR3 inhibition, as reflected in the burst surprise values (Supplementary Fig. 4a). These observations indicated that TACR3 activity is essential in controlling spine density and that it influences the dynamic activity correlations within neuronal networks. Furthermore, TACR3 manipulation can induce modifications in neuronal firing patterns, emphasizing its importance in shaping the overall activity in neural circuits.

### Aberrant dendritic TACR3 drives spine shrinkage and pruning

We were interested in deciphering the effect of TACR3 overexpression on synaptic function and structure. An immunocytochemical study of TACR3 was undertaken in primary hippocampal neurons expressing EGFP, and TACR3-positive punctae were seen around dendrites. However, when voxels around the dendrites and spines were excluded, no discernable TACR3 was evident within dendrites (Fig. 5g), indicating this receptor is not expressed on dendrites and spines. To corroborate this observation, synaptosomal fractionation was used to pinpoint the precise neuronal location of endogenous TACR3, revealing this receptor accumulated in the presynaptic compartment, but it was excluded from the postsynaptic density (PSD: Fig. 5h). The accuracy of the fractionation was confirmed using well-established pre- and postsynaptic markers as controls (PSD-95 and synaptophysin: Fig. 5h).

Subsequently, we incorporated TACR3 into the pSinRep5/Sindbis expression system along with IRES-EGFP (Fig. 5i, top panel), an approach that preserves the natural sequence and function of TACR3 while enabling the infected neurons to be visualized. TACR3 overexpression was confirmed through RT-PCR analysis (Fig. 5i, left panel). When the pCaMKII (T286)/tCaMKII fluorescence was measured on a microplate reader, TACR3 overexpression dampened CaMKII phosphorylation (Fig. 5i, right panel) in contrast to the increase in pCaMKII when TACR3 was inhibited. This finding strengthened the idea that TACR3 modulates CaMKII activity. Nonetheless, global activation of the PKC pathway is not observed upon TACR3 overexpression, as verified through Western blotting with the use of phospho-PKC substrate antibodies (Supplementary Fig. 5a). When the distribution of endogenous and recombinant TACR3 was examined by immunocytochemistry (Fig. 5j, k), TACR3 overexpression led to the presence of TACR3 in dendrites. At a higher resolution, we observed native TACR3 surrounding the dendrites (indicated by purple dots, Fig. 5k), while some receptor was also evident within dendrites and spines following TACR3 overexpression (indicated by blue dots, Fig. 5k–l). This aberrant expression of TACR3 (see Fig. 5l for TACR3 expression in spines) was associated with a reduced spine density and smaller spine sizes (Fig. 5m–o). Hence, the aberrant expression of TACR3 can significantly affect synaptic structure and thereby influence neuronal activity.

### Testosterone mitigates LTP deficiencies in neurons with malfunctioning TACR3

**Fig. 6:**
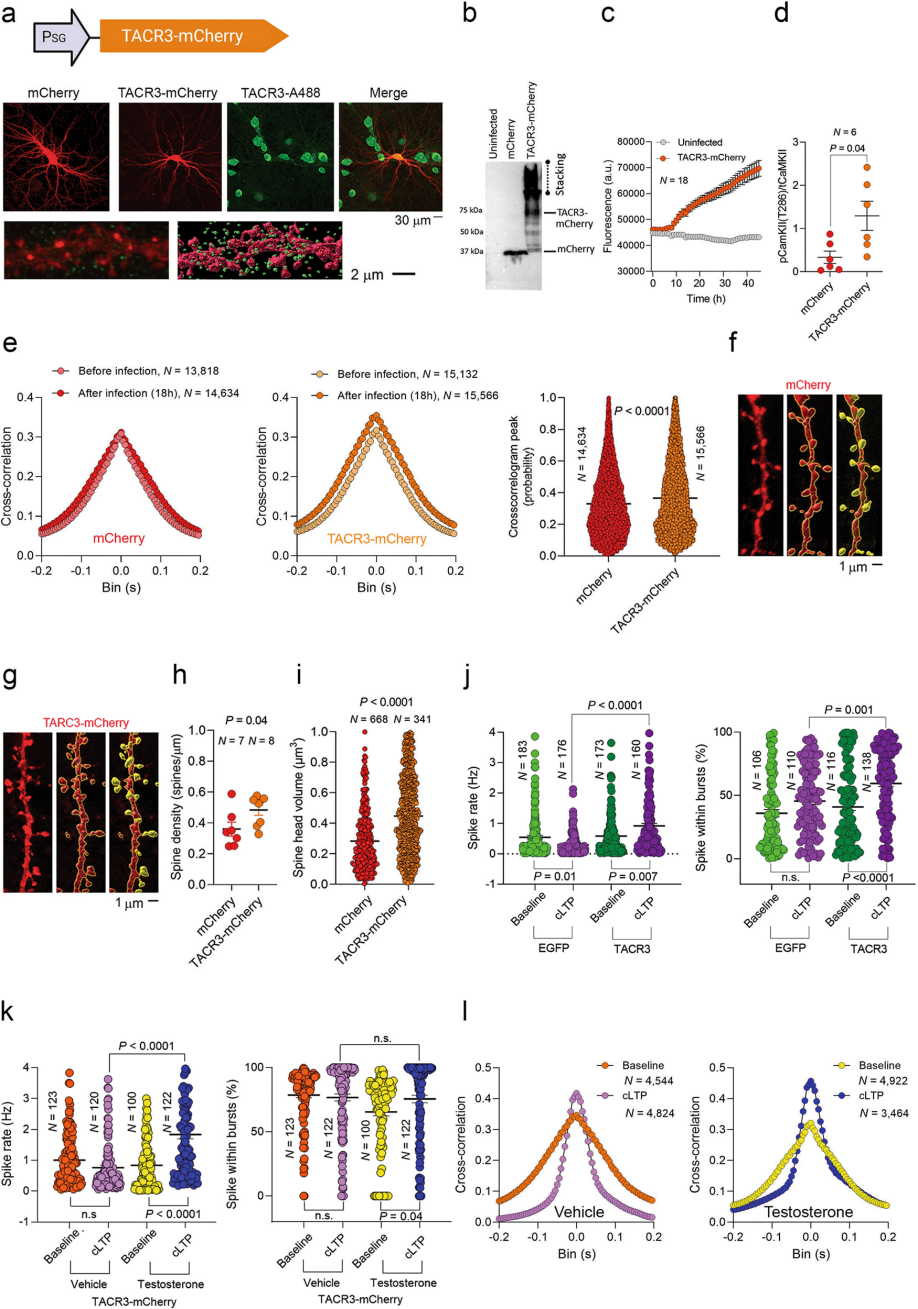
Testosterone treatment rescues the impaired response to cLTP induction observed in neurons with dysfunctional TACR3 expression. **a** *Top*: Structure of the TACR3-mCherry overexpression construct. *Middle*: Representative confocal images of neurons expressing mCherry or TACR3-mCherry (red) immunostained with a TACR3 antibody (green). *Bottom*: High-magnification view of a dendrite from a TACR3-mCherry expressing neuron (left), revealing TACR3 puncta surrounding the dendrite (depicted as green circles) and the three-dimensional reconstruction of the dendrites, spines and TACR3 puncta obtained with the Imaris software (right). **b** Western blot analysis illustrating the migration of mCherry on the gel compared to the delayed migration of TACR3-mCherry and its retention in the stacking gel, indicative of the aggregation of this fusion protein. **c** Real-time monitoring of TACR3-mCherry fluorescence measured using a microplate reader over 48 h. *N* represents the number of cultures. **d** Phospho CaMKII/total CaMKII levels in neuorns infected with mCherry or with TACR3-mCherry. *N* is the number of culturs and the *P* value was determined with a Mann-Whitney test. **e** *Left*: The effect of mCherry and TACR3-mCherry on average cross-correlograms obtained from spike recordings subjected to spike sorting. The average cross-correlograms before infection and 18 h after infection highlight the changes in neuronal correlation following the expression of TACR3-mCherry. *N* represents the number of neuron pairs analyzed. *Right*: The impact of recombinant protein expression on the peaks of cross-correlograms. Each data point on the graph represents the peak value of an individual cross-correlogram between two neurons, and the statistical significance was assessed using a Mann-Whitney test. **f**, **g** *Left*: Dendrites from neurons expressing mCherry alone (mCherry) or neurons overexpressing the TACR3-mCherry fusion protein. *Middle*: Three-dimensional structure of the same dendrites captured using the Surface module of the Imaris software. *Right*: Dendritic spine heads were visualized using the Surface module of the Imaris software, allowing the spine density and spine head volume to be quantified. **h**, **i** Quantification of spine density and spine head volume using Imaris software. Statistical significance was determined with a Mann-Whitney test, with *N* representing the number of dendrites analyzed for spine density or the number of spines analyzed for spine head volume. Each data point represents the value obtained from a single dendrite or spine. **j**, **k** Spike rate and the proportion of the spikes within bursts in neurons expressing EGFP, TACR3 or TACR3-mCherry, before and after cLTP induction, and with or without testosterone pre-treatment. Each dot represents the value of a single neuron, and the statistical significance was determined using a Kruskal-Wallis test followed by Dunn’s multiple comparisons tests. *N* represents the number of neurons. **l** The impact of cLTP induction and testosterone pre-treatment on the cross-correlograms of neurons expressing TACR3-mCherry. Average cross-correlograms were analyzed before cLTP induction and 4 h after induction. The number of neuron pairs analyzed is represented by *N*. For the raster plots of this experiment and the cross-correlograms of neurons expressing mCherry, please refer to Supplementary Figs. 5b and 6a.

We next generated a TACR3-mCherry fusion protein and expressed it in primary neurons using the Sindbis vector (Fig. 6a, top panel), using the expression of mCherry alone as a control. This fusion protein could be detected in neurons (Fig. 6a, lower panels) but it aggregated intracellularly, as also evident in western blots (Fig. 6b). TACR3 overexpression was confirmed by real-time fluorescent quantification in a microplate reader (Fig. 6c), and similarly to the effect of osanetant we observed an increase in the pCaMKII in neurons expressing this recombinant protein and in the level of phosphorylated PKC substrates (Fig. 6c and Supplementary Fig. 5a, respectively). To test the functionality of TACR3-mCherry, we assessed the activity of the infected neurons on MEAs, and increased cross-correlation was evident, aligning with the effects of osanetant (Fig. 6e). We also observed lower dendritic spine density (Fig. 6f–h), mirroring the osanetant effect, and in contrast to the impact of the functional TACR3 (TACR3-IRES-EGFP), an expansion in spine head volume was seen (Fig. 6i). Hence, the TACR3-mCherry fusion protein demonstrated dysfunctionality and its behavior was akin to the pharmacological inhibition of TACR3.

Treating males with CHH primarily involves testosterone administration through hormone replacement therapy to restore the hormonal balance [73]. In this context, we explored whether testosterone treatment could address the functional deficiencies observed in neurons expressing TACR3-mCherry. TACR3 deficiency in SA rats or its inhibition with osanetant impaired LTP and to investigate this further, we induced cLTP in neurons overexpressing different recombinant proteins: EGFP, TACR3, mCherry, or TACR3-mCherry (Supplementary Fig. 5b). The induction of cLTP increased the firing rate and proportion of spikes within bursts in EGFP-expressing neurons (Fig. 6j), with an even more pronounced effect in neurons overexpressing functional TACR3 (TACR3-IRES-EGFP) reflecting the influence of TACR3 activity on LTP (Fig. 6j). By contrast, neurons expressing dysfunctional TACR3 (TACR3-mCherry) did not display these changes when cLTP was induced (Fig. 6k). Remarkably, a 1-h pre-treatment with testosterone (10 nM) rescued the response of TACR3-mCherry-expressing neurons to cLTP (Fig. 6k). Moreover, cLTP induction in mCherry-expressing neurons resulted in narrower cross-correlograms with a 50% increase in peak height (Supplementary Fig. 6a), which was further elevated to 65% after testosterone pre-treatment (Supplementary Fig. 6a). Importantly, TACR3-mCherry expression significantly reduced this peak increase to 17%, although testosterone pre-treatment restored this to levels comparable to non-TACR3-mCherry expression (45%: Fig. 6l). These findings highlight the ability of testosterone to correct deficits in LTP expression caused by dysfunctional TACR3.

To delve deeper into the mechanisms by which testosterone may counteract the effects of TACR3 deficiency, we administered either osanetant (100 nM), testosterone (10 nM), or a combination of both drugs to neurons. Consistent with previous observations, osanetant increased spine density, and while testosterone did not affect spine density, it was unsuccessful in restoring it to normal levels when combined with osanetant. These findings suggest that testosterone mitigates the detrimental effects of TACR3 deficiency by functionally correcting firing patterns and plasticity, rather than by altering spine density (Supplementary Fig. 6a, b).

## Discussion

The present study sets out to explore the intricate connection between TACR3, testosterone, and anxiety, the latter a prominent phenotype observed in individuals with non-syndromic normosmic CHH. These experiments were prompted by the observation of weaker TACR3 expression in the ventral hippocampus of rats exhibiting elevated anxiety (SA rats). This intriguing finding led us to look closely at the synaptic and molecular pathways that link TACR3 deficiency, anxiety, sex hormones and plasticity.

Anxiolytic effects of TACR3 agonists and anxiogenic effects of TACR3 antagonists have already been reported [21, 74–76], and TACR3 expression has been found to vary in the amygdala relative to the state of anxiety displayed by rats [77]. However, the significance of TACR3 in anxiety regulation has remained unexplored, particularly in the context of sex hormone function and at the molecular and synaptic levels. The findings of this study shed light on the potential importance of hippocampal TACR3 in modulating anxiety, providing valuable insight into the intricate interplay between TACR3, sex hormones, and anxiety-related mechanisms. We studied the bidirectional association between testosterone and TACR3, focusing on the hippocampal formation. The ventral hippocampus, known for its involvement in anxiety-related processes, is crucial in modulating anxiety-like behavior [41, 54, 78]. We found that TACR3 expression in the hippocampus increases in conjunction with sexual maturation, and it is influenced by sex hormones, even in adulthood. Interestingly, male adolescent rats with lower testosterone levels display more intense anxiety-like behavior than adult males, while systemic testosterone administration increased hippocampal TACR3 expression. These findings suggest a potential role for testosterone in modulating the effects of TACR3 on anxiety. Notably, administration of the TACR3 inhibitor osanetant decreased serum testosterone, indicating mutual regulation between TACR3 and testosterone.

The control of serum testosterone levels by TACR3 is consistent with its known role in hypogonadism, whereas the discovery that sex hormones influence TACR3 expression in the hippocampus was a more unexpected finding. Sex hormones are integral to multifaceted networks involving various organs and systems, such as the HPG axis, and they are pivotal in their regulation. Our study assessed how testosterone and other sex hormones influenced hippocampal TACR3 expression, revealing intricate interactions that potentially have ramifications for understanding anxiety-like behavior and synaptic plasticity. This relationship could possibly be explained by a feedback loop in which TACR3 not only regulates sex hormones but is also affected by them. Such a system might create a dynamic equilibrium whereby minor alterations to one element could have important consequences on the other, possibly even involving additional signaling molecules or pathways not directly explored here. Revealing these complex relationships between sex hormones and TACR3 expression deepens our grasp of the subtle and multifaceted effects of the estrous cycle and of testosterone on TACR3 regulation. It further highlights the existence of sex-specific effects, emphasizing that individual hormonal makeup could influence anxiety disorders. Understanding the intricate connection between the estrous cycle, sex hormones, and TACR3 might identify new avenues for gender-targeted therapeutic approaches.

The impact of TACR3 on dendritic spines and the involvement of the PKC and CaMKII signaling in regulating TACR3-related synaptic plasticity was also explored. TACR3 deficiency or dysfunction increases the spine density, while aberrant TACR3 expression in spines causes spine shrinkage and pruning. The CaMKII pathway was activated following TACR3 inhibition with osanetant, leading to an increase in spine density in primary hippocampal neurons [28, 79]. This elevation is a compelling indicator of the profound engagement of TACR3 in the dynamics of CaMKII phosphorylation. This is particularly noteworthy as the activated, phosphorylated form of CaMKII impedes further induction of LTP, a phenomenon reminiscent of the observations from SA rats in vivo. Conversely, senktide does not notably affect pCaMKII levels independently, but it boosts CaMKII phosphorylation when combined with cLTP induction. Previous studies demonstrated that increased CaMKII activity triggers LTP to its maximum potential, thereby impeding any further increase in LTP. In other words, postsynaptic CaMKII activity plays a pivotal role in potentiating synaptic transmission, and it is both necessary and sufficient to generate LTP [60]. Consequently, the co-administration of osanetant and glycine does not provoke a strong upregulation of pCaMKII, as seen with osanetant alone, because the postsynaptic expression of constitutively active CaMKII impedes further LTP induction. CaMKII enhances synaptic transmission by phosphorylating postsynaptic GluA1, influencing its role in LTP regulation.

The observation that the phosphorylation of CaMKII is affected by osanetant pretreatment and inhibited by glycine-induced cLTP aligns with the measurement of AMPAR surface expression with FORTIS. Specifically, osanetant enhances AMPAR surface expression to a similar extent as LTP, while cLTP induction under osanetant treatment dampens AMPAR surface expression. CaMKII-mediated phosphorylation of GluA1 at S831 had a significant influence on LTP regulation by increasing channel conductance and enhancing the trafficking of AMPARs to synapses [28]. This phosphorylation has been shown to be involved in modulating the mechanisms underlying LTP. These results further support the notion that osanetant and cLTP have similar effects on CaMKII phosphorylation and AMPAR surface expression, enhancing our understanding of the mechanisms regulating synaptic plasticity.

The DG is associated with affective processing and innate anxiety [53], and TACR3 deficiency in rats provoked an increase in spine density and basal synaptic transmission in this structure. This heightened connectivity between the DG and the entorhinal cortex may contribute to abnormal sensory processing and emotion dysregulation, potentially playing a role in anxiety disorders [80–82]. Furthermore, TACR3 deficiency or inhibition was seen to impair LTP since in vivo experiments on SA rats with little hippocampal TACR3 were unable to express LTP in the DG. LTP can be impaired without a decrease in spine density [83]. Indeed, establishing a straightforward correlation between spine density and LTP is complex due to the multifaceted nature of LTP substrates, which encompass molecular factors within synapses rather than being solely confined to dendritic spine density. However, it is essential to note that the rise in spine density alongside a blockade of LTP in SA rats may be connected to TACR3 deficiency. The effect of osanetant is also worth considering as it increases spine density and inhibits cLTP in cultured neurons. This inhibition, similar to TACR3 deficiency in SA rats, highlights TACR3’s role in regulating both spine density (in vitro and in vivo) and LTP dynamics. We suggest that TACR3 activity is intrinsically involved in the sculpting of LTP and the absence of this activity in SA rats offers a compelling explanation for their inability to manifest LTP in an in vivo setting.

Finally, we studied the dysfunctional characteristics of the TACR3-mCherry fusion protein, resembling the pharmacological inhibition of TACR3. While functional TACR3 promotes LTP, neurons expressing TACR3-mCherry do not respond correctly to LTP induction. Notably, testosterone pre-treatment rescues the impaired response of TACR3-mCherry expressing neurons to cLTP, indicating the therapeutic potential of testosterone in correcting LTP deficits caused by dysfunctional TACR3. These findings have implications for the development of treatment strategies targeting CHH and anxiety.

To summarize, our study proposes TACR3 as a critical link between LTP and anxiety, providing insights into the mechanisms underlying anxiety, and potential treatments for anxiety disorders involving testosterone and the induction of plasticity.

### Supplementary information

Supplemental material

Supplementary Figure 1

Supplementary Figure 2.

Supplementary Figure 3.

Supplementary Figure 4.

Supplementary Figure 5.

Supplementary Figure 6.

## Data Availability

The complete dataset is publicly available in a designated repository (10.5281/zenodo.8305270).

## Code availability

The scripts used for MEA analysis are available upon request.
