# Supplementary material for: Interplay between hippocampal TACR3 and systemic testosterone in regulating anxiety-associated synaptic plasticity
Source: Mol Psychiatry. 2023 Dec 22;29(3):686–703. doi: 10.1038/s41380-023-02361-z (PMC11153148; doi:10.1038/s41380-023-02361-z)
Supplement: Supplementary file 1 — Supplemental material [file 41380_2023_2361_MOESM1_ESM.docx]

**Supplementary Materials and Methods**

***Hippocampus dissection***

The rats were sacrificed, and their brains were removed, dissecting out the hippocampus and snap-freezing the tissue by immersion in liquid nitrogen. The samples were stored at -80 °C until further use.

***Microarray analyses***

RNA was extracted from SA and MA rats (N = 4 per group) using the PureLink™ RNA Mini Kit (Thermo Fisher, #12183018A), and the quality of the RNA was confirmed in electropherograms on an Agilent 2100 Bioanalyzer (Suppl. Fig. 1). The RNA concentration ranged from 57 to 248 ng/ml, and biotin-labeled cDNA was produced from this total RNA (4 µg) using One-Cycle Target Labeling and Control Reagents (Affymetrix). Fragmented and denatured cDNA preparations (10 µg) were hybridized to the Affymetrix Rat Genome 430 2.0 GeneChip array, and the microarrays were washed and stained with streptavidin-phycoerythrin, prior to scanning at 1.56 µm resolution using a GeneChip Scanner 3000 7G System (Affymetrix). Raw intensity values were normalized using the Robust Multi-array Analysis (RMA) algorithm and after data processing, the probes were tested for changes in expression using empirical Bayes-moderated t-statistics.

***Western Blotting***

Cell plates were washed once with ice-cold PBS before they were lysed by scrapping in lysis buffer (10 mM HEPES [pH 7.4], 150 mM NaCl, 10 mM EDTA, 1 % Triton X-100, protease inhibitor cocktail "Complete mini EDTA-free" and phosphatase inhibitor cocktail "PhosSTOP": Roche, 04693159001 and 04906837001). Lysates were collected and centrifuged at 500 xg for 5 minutes at 4 °C, and the protein in the supernatants was quantified. Protein concentration was determined using the BCA protein assay (Pierce, 23227) and with known bovine serum albumin (BSA) concentrations as standards to determine the concentration of the protein extracts. Equal amounts of protein were prepared in 4x sample loading buffer and the samples were boiled at 95 °C for 5 minutes immediately prior to electrophoresis. Proteins (10-40 µg per lane) were separated according to their molecular weight by SDS-PAGE in a MiniPROTEAN Tetra Cell Vertical Electrophoresis system (Bio-Rad) and in 1x running buffer (25 mM Tris [pH 8.3], 192 mM glycine, 0.1% SDS in dH_2_O). Following electrophoresis, the proteins were transferred in transfer buffer (25 mM Tris [pH 7.6], 192 mM glycine, 20% methanol in dH_2_O) from the gel to a PVDF membrane (pore size 0.45 µm: Amersham Hybond, 10600023) for 90 minutes at 400 mA using the Mini Trans-Blot Cell transfer system (Bio-Rad). The membrane was then stained with Ponceau S (Sigma Aldrich, P3504) to confirm successful protein transfer, and the membranes were blocked for 1 hour at room temperature (RT) with gentle shaking with 5 % (w/v) non-fat dry milk powder in TBS-T (20 mM Tris [pH 7.6], 137 mM NaCl, 0.1% Tween-20 in dH_2_O). To detect phosphorylated proteins, 5 % (w/v) Phospho BLOCKER™ Blocking Reagent in TBS-T was used (Cell Biolabs, AKR-103). The membranes were probed with specific primary antibodies diluted in blocking solution overnight at 4 °C with gentle shaking, washed three times for 5 minutes with TBS-T and then incubated with the corresponding horseradish peroxidase (HRP)-labeled secondary antibody in blocking solution for 1 hour at RT. The membranes were again washed as described above and the antibodies bound to their target proteins were detected using enhanced chemiluminescence (Luminata Forte Western HRP Substrate, Millipore, MIWBLUF0100 or CYANAGEN # XLS142,0250) in a MyECL Imager (Thermo Scientific) or iBright™ FL1500 Imaging System, and using iBright™ analysis software, Fiji or Quantity One software (Bio-Rad).

*Treatment with senktide and osanetant for western blot analysis.* Rat hippocampal neurons at 18 DIV were exposed to growth medium containing senktide (Tocris, cat. no. 1068) or osanetant (Axon MedChem, cat. No. 1533) diluted to a final concentration of 100 nM for 4 hours. Subsequently, the neurons were washed twice with pre-equilibrated bathing buffer (129 mM NaCl, 4 mM KCl, 10 mM Hepes [pH 7.4], 10 mM glucose, 18 mM sodium bicarbonate, 4 mM CaCl_2_) in the presence of the drugs and they were then incubated for 30 min in bathing buffer containing 0.02 mM bicuculine, 0.001 mM strychnine and 0.2 mM glycine to induce cLTP. The cells were then lysed in RIPA buffer containing a Complete™ EDTA-free Protease Inhibitor Cocktail (Roche) and phosphatase inhibitors (PhosSTOP: Roche).

*Western Blots*. Total protein (20-40 μg) was resolved on 10% Criterion™ TGX™ Precast Midi Protein Gels (Bio-Rad) and transferred to PVDF membranes (Amersham Hybond, cat. no. 10600023) by semi-dry Fast Blotter transfer (Thermo Scientific). The membranes were blocked with 5% PhosphoBlocker™ Blocking Reagent (Cell Biolabs, cat. no. AKR-104) in TBS-T and probed with antibodies against pCamKII (Thr286: Sigma Aldrich, cat. no. 05-533) and PKC substrates (Cell Signalling Technology, cat. no. 2261S) or the CaMKII antibody (Sigma Aldrich, cat. no. C6974-.2ML) diluted in 5% Blotto, non-fat dry milk (Santa Cruz, cat. no. Sc-2325) and TBS-T. The secondary HRP conjugated antibody (Cell Signalling Technology, cat. no. 7074S or 7076S) was diluted in either PhosphoBlocker™ Blocking Reagent or Blotto, non-fat dry milk, and antibody binding was visualized with Immobilion Forte Western HRP substrate (Milipore, cat. no. WBLUF0500) and on an iBright™ CL1500 Imaging System. ImageJ software was used for quantification.

A Cavalieri volume estimation was performed to determine the volume of the dentate gyrus using stereo investigator software (MicroBrightField, Williston, VT) and blind to the rats' experimental performance. The volume of each subfield was estimated based on a one-in-four systematic random series of 50 μm Nissl-stained sections using a 200 μm^2^ point-counting grid. A range of 350 to 500 grid points from 6 to 16 sections was counted for each dentate gyrus, resulting in coefficient of error (CE) estimates of <0.1.

***Immunocytochemistry***

For immunofluorescence experiments, hippocampal neurons were seeded on 12 mm diameter coverslips (Menzel-Gläser, CB00120RA1), fixed in 4% paraformaldehyde (PFA) in PBS for 10 minutes at RT, and washed three times with PBS. The cells were then incubated in blocking solution (3% BSA, 5 % goat serum, 0.1 % Triton X-100 in PBS) for 1 hour at RT and then probed overnight at 4 °C with specific primary antibodies diluted in blocking solution. After washing three times for 5 minutes with PBS, the cells were then incubated with the corresponding fluorochrome-conjugated secondary antibodies in a blocking buffer for 1 hour at RT. Finally, the cells were mounted using Prolong Gold Antifade Reagent (Thermo Fisher Scientific, P36934) and images were acquired on a ZEISS LSM 900 with Airyscan 2, using ZEN (ZEISS Efficient Navigation software) and two different objectives: LD Plan-NEOFLUAR 20x/0.4 Corr and LD LCI Plan-APOCHROMAT 63x/1.2 with immersion oil (ImmersolTM 518F, Zeiss). Single field images of 1024 x 1024 pixels were collected using a 488 nm laser. Multiple stage positions were collected using a motorized stage with a step size of 5-10 μm.

***Synaptosomal fractionation***

Synaptosomes were isolated from the brain hippocampus or cortex in 1 ml of buffer A: 320 mM sucrose, 10 mM HEPES [pH 7.4], a 1:100 protease inhibitor cocktail, and PhoSTOP tablets (used according to manufacturer instructions: Roche). The tissue samples were homogenized with six strokes in a Douncer homogenizer and then passed through a 25G needle six times to obtain a total lysate fraction. The remaining volume was centrifuged at 1400 xg for 10 minutes at 4 ºC and the supernatant recovered, while the pellet was re-homogenized and centrifuged again under the same conditions. The supernatants were combined and centrifuged at 13,000 xg for 15 minutes at 4 ºC, and the resulting pellets containing the synaptosomes were resuspended in 1 ml of Buffer A and centrifuged again under the same conditions to obtain the crude synaptosomal fraction. This fraction was then resuspended in 400 µl of buffer B (10 mM HEPES [pH 8], 1% Triton X-100) and incubated for 30 minutes at 4 ºC on a 360º rotor. The samples were centrifuged again at 21,000 xg for 15 minutes at 4 ºC, and the supernatant obtained represented the pre-synaptic fraction. The pellet was resuspended in 200 µl of buffer B and centrifuged at the same conditions. The resulting supernatant was removed completely, and the pellet obtained was considered the post-synaptic fraction.

***Neuronal Cultures***

Primary hippocampal/cortical neurons were prepared from rats as described elsewhere^1-3^. In brief, neurons were dissociated from E18 embryos and plated in 96-well optical plates. The neurons were maintained at 37 °C in a 5% CO_2_ incubator and 20% of the medium was changed every 7 days. A similar protocol was followed for small-scale cultures in 12-24-well plates on poly-L-lysine-coated coverslips. Cultures were treated with cytosine β-D-arabinofuranoside (final concentration 5 μM: Sigma-Aldrich, C1768) to diminish glial proliferation (DIV 7), and peripheral wells were loaded with water to prevent edge effects due to thermal gradients and differential evaporation rates^4^.

**Supplementary Figure Legends**

**Supplementary Figure 1. The integrity of RNA from MA, IA and SA rats.**

***Left:*** RNA Integrity Number (RIN): RIN values were generated based on the electrophoretic profiles (left), providing an objective measure of RNA integrity. The RIN scale ranges from 1 (completely degraded) to 10 (intact). ***Right:*** Representative gel image showing the migration of RNA samples with intact 28S and 18S ribosomal RNA (rRNA) bands.

**Supplementary Figure 2. Transfection of BHK cells for Sindbis virus production.**

Brightfield and fluorescence images depicting BHK cells transfected with RNA for the production of Sindbis virus. The RNA used encoded TACR3-IRES-EGFP or TACR3-mCherry, enabling the expression of the respective recombinant proteins. The images provide visual evidence of successful transfection and expression of the protein, confirming the successful production of Sindbis virus.

**Supplementary Figure 3. Dentate gyrus volume and spine density in the lateral amygdala.**

**a.** Outcomes from the Elevated Plus Maze (EPM) behavioral assessments following 10 days of intracerebroventricular osanetant administration were analyzed. Statistical significance was determined using the Mann-Whitney test.

**b.** Western blots probed for phospho CaMKII (T286) following cLTP induction.

**c.** Cavalieri volume estimation was performed using stereo investigator software to determine the dentate gyrus volume. Sections were sampled systematically and analyzed using a point-counting grid, resulting in accurate estimates of volume with a low coefficient of error (CE). Each dot represents the value for a single rat.

**d.** ***Left,*** representative confocal images of dendrites from pyramidal-like neuron in the lateral amygdala. ***Right,*** Spine density in the lateral amygdala was compared between severely (SA) and moderately anxious (MA) rats using Sholl analysis. The analysis revealed no significant differences in spine density.

**Supplementary Figure 4. Effects of TACR3 manipulation on firing patterns in a multielectrode array.**

Graphs depict the responses of neurons treated with osanetant or senktide, revealing distinct changes in firing patterns relative to untreated neurons. The number of neurons (*N*) is indicated, and the statistical significance was determined using a two-way ANOVA.

**Supplementary Figure 5. Phospho-PKC substrates and raster plots.**

**a.** Western blot of phospho PKC substrates in cultures expressing TACR3 and mCherry-TACR3. N represents the number of cultures, with each dot representing the value of a single culture and the data also presented as the mean ± SEM. Statistical significance was determined using a Kruskal-Wallis test, followed by Dunn's multiple comparisons tests, and N represents the number of cultures in each group.

**b.** Raster plots illustrating the firing activity of neurons in multielectrode array recordings from mCherry and TACR3-mCherry expressing neurons, with or without testosterone pre-treatment and cLTP induction: black lines represent individual electrode activity; green lines indicate the bursting activity of specific electrodes; and purple rectangles depict network bursts. The electrode numbers are displayed on the left side of the plots.

**Supplementary Figure 6. The effect of testosterone and osanetant on the cross-correlation and spine density.**

**a.** The impact of cLTP induction, with or without testosterone pre-treatment, on the average cross-correlograms obtained from spike recordings that underwent spike sorting. Cross-correlograms were computed to assess the correlation between pairs of neurons based on their spike times.

**b. *Left:*** Dendrites from neurons expressing EGFP and treated with osanetant, testosterone or both. ***Right:*** Three-dimensional structure of the same dendrites captured using the Surface module of Imaris software, visualizing dendritic spine heads and quantifying the spine density.

**c.** Quantification of spine density and spine head volume using Imaris software. *N* represents the number of dendrites analyzed for spine density and each data point represents the value obtained from a single dendrite. The statistical significance was determined using a Kruskal-Wallis test followed by Dunn's multiple comparisons tests.

**References**

1. Knafo S, Sanchez-Puelles C, Palomer E, Delgado I, Draffin JE, Mingo J *et al.* PTEN recruitment controls synaptic and cognitive function in Alzheimer's models. *Nat Neurosci* 2016; **19**(3)**:** 443-453.

2. Calleja-Felipe M, Wojtas MN, Diaz-González M, Ciceri D, Escribano R, Ouro A *et al.* FORTIS: a live-cell assay to monitor AMPA receptors using pH-sensitive fluorescence tags. *Translational psychiatry* 2021; **11**(1)**:** 1-13.

3. Sánchez-Puelles C, Calleja-Felipe M, Ouro A, Bougamra G, Arroyo A, Diez I *et al.* PTEN Activity Defines an Axis for Plasticity at Cortico-Amygdala Synapses and Influences Social Behavior. *Cereb Cortex* 2020; **30**(2)**:** 505-524.

4. Lundholt BK, Scudder KM, Pagliaro L. A simple technique for reducing edge effect in cell-based assays. *Journal of biomolecular screening* 2003; **8**(5)**:** 566-570.
