# Supplementary figures and images for: Interplay between hippocampal TACR3 and systemic testosterone in regulating anxiety-associated synaptic plasticity
Source: Mol Psychiatry. 2023 Dec 22;29(3):686–703. doi: 10.1038/s41380-023-02361-z (PMC11153148; doi:10.1038/s41380-023-02361-z)

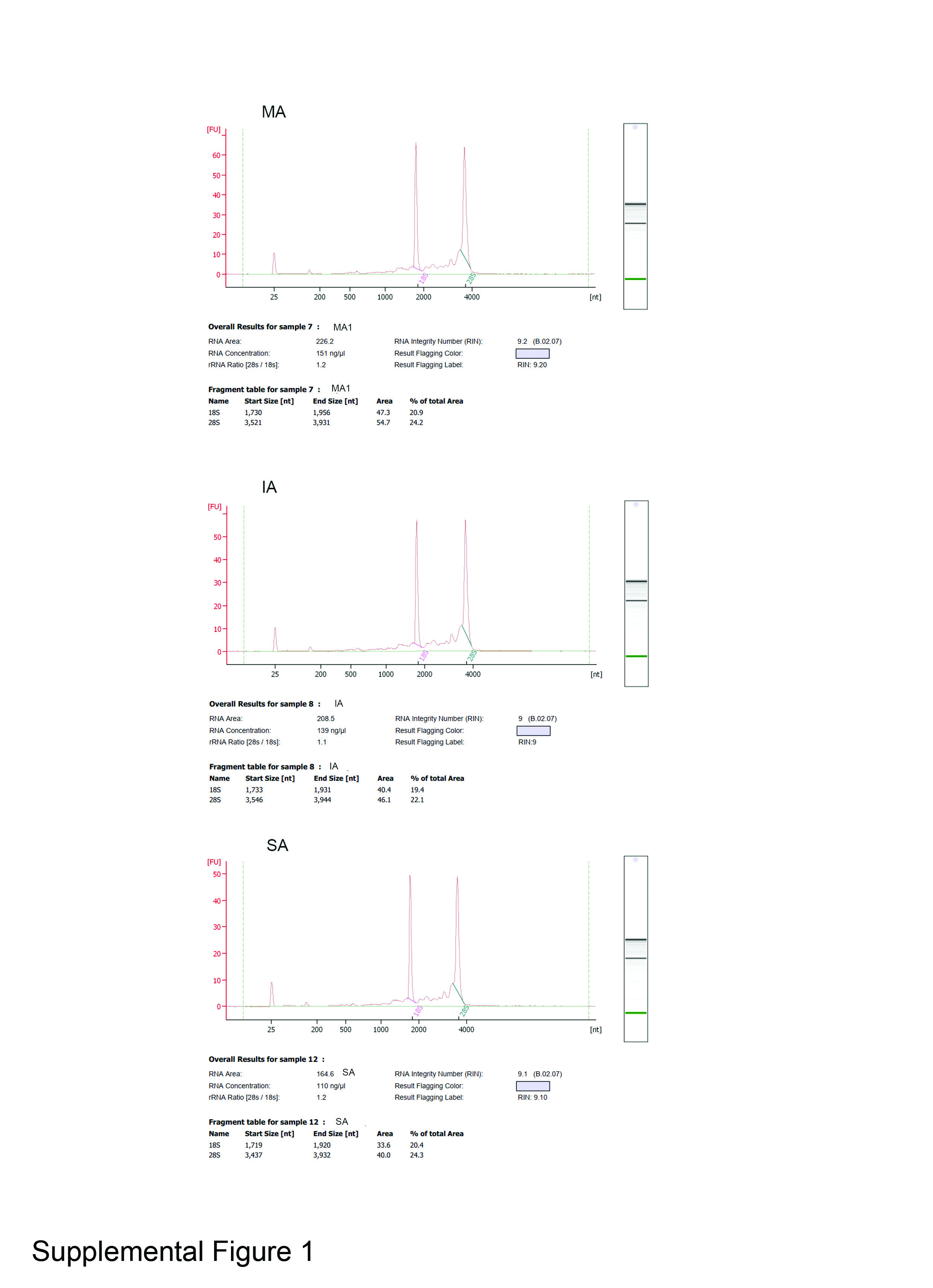

Supplement: Supplementary file 2 — Supplementary Figure 1 [file 41380_2023_2361_MOESM2_ESM.jpg]

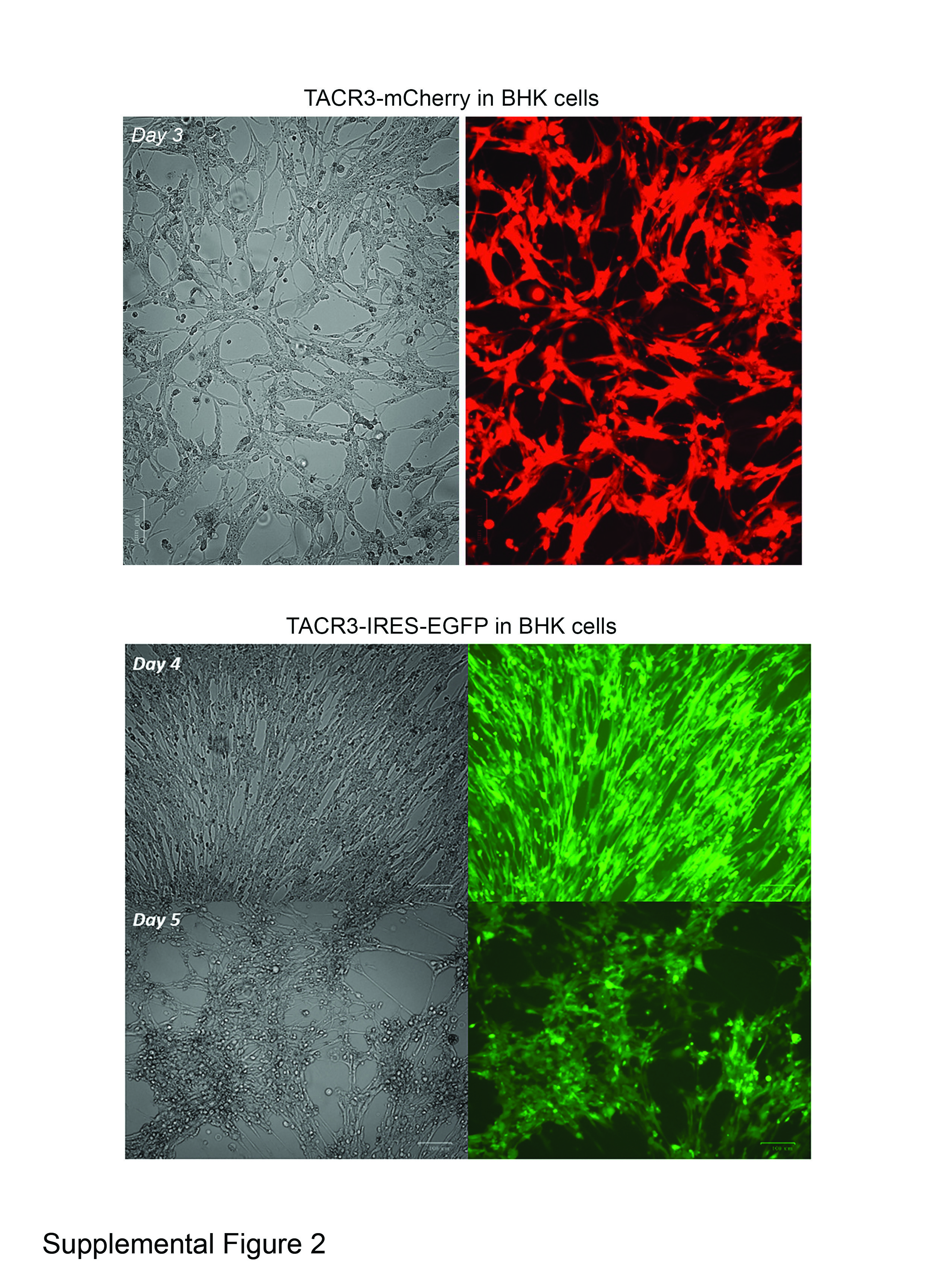

Supplement: Supplementary file 3 — Supplementary Figure 2. [file 41380_2023_2361_MOESM3_ESM.jpg]

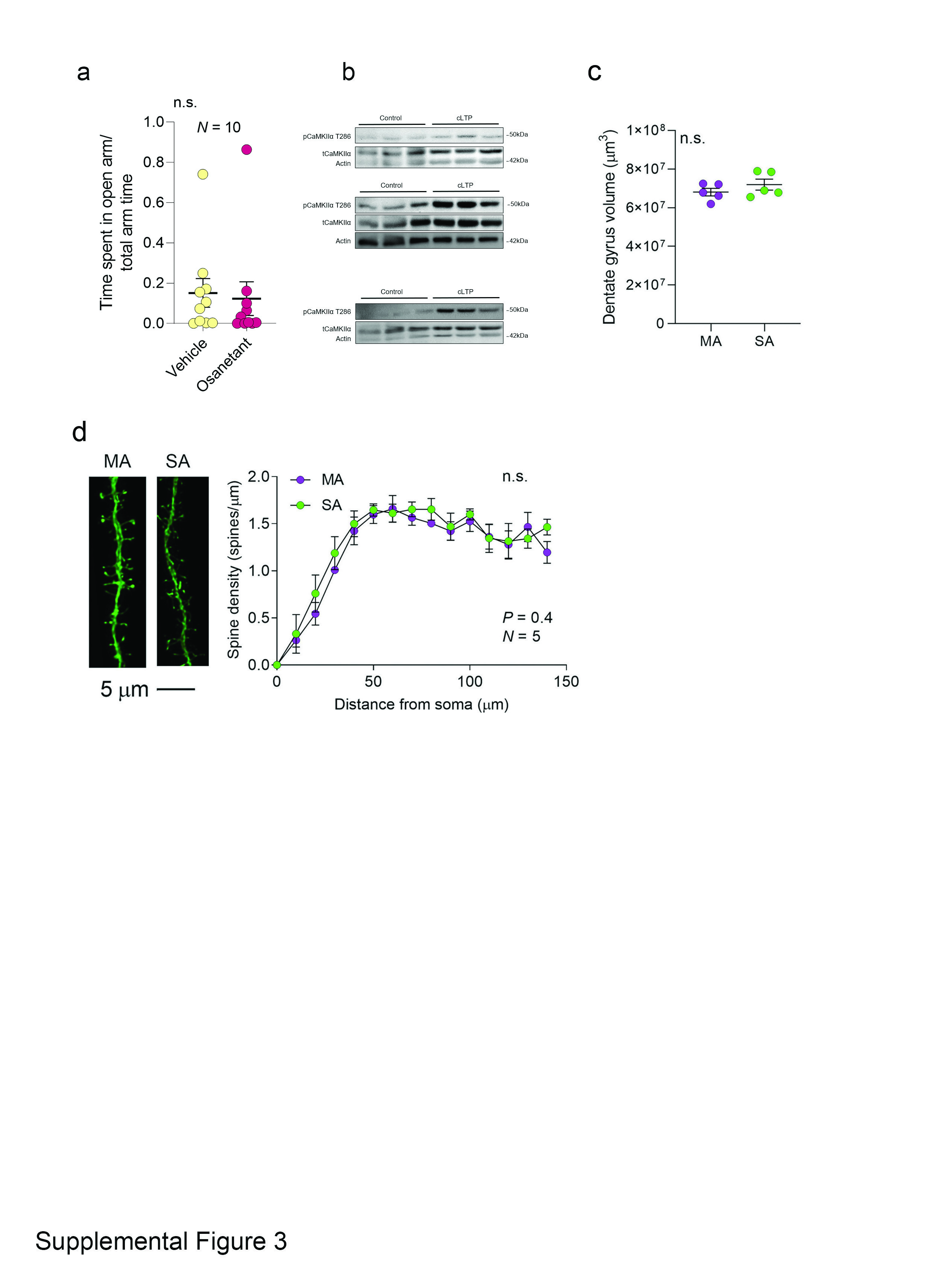

Supplement: Supplementary file 4 — Supplementary Figure 3. [file 41380_2023_2361_MOESM4_ESM.jpg]

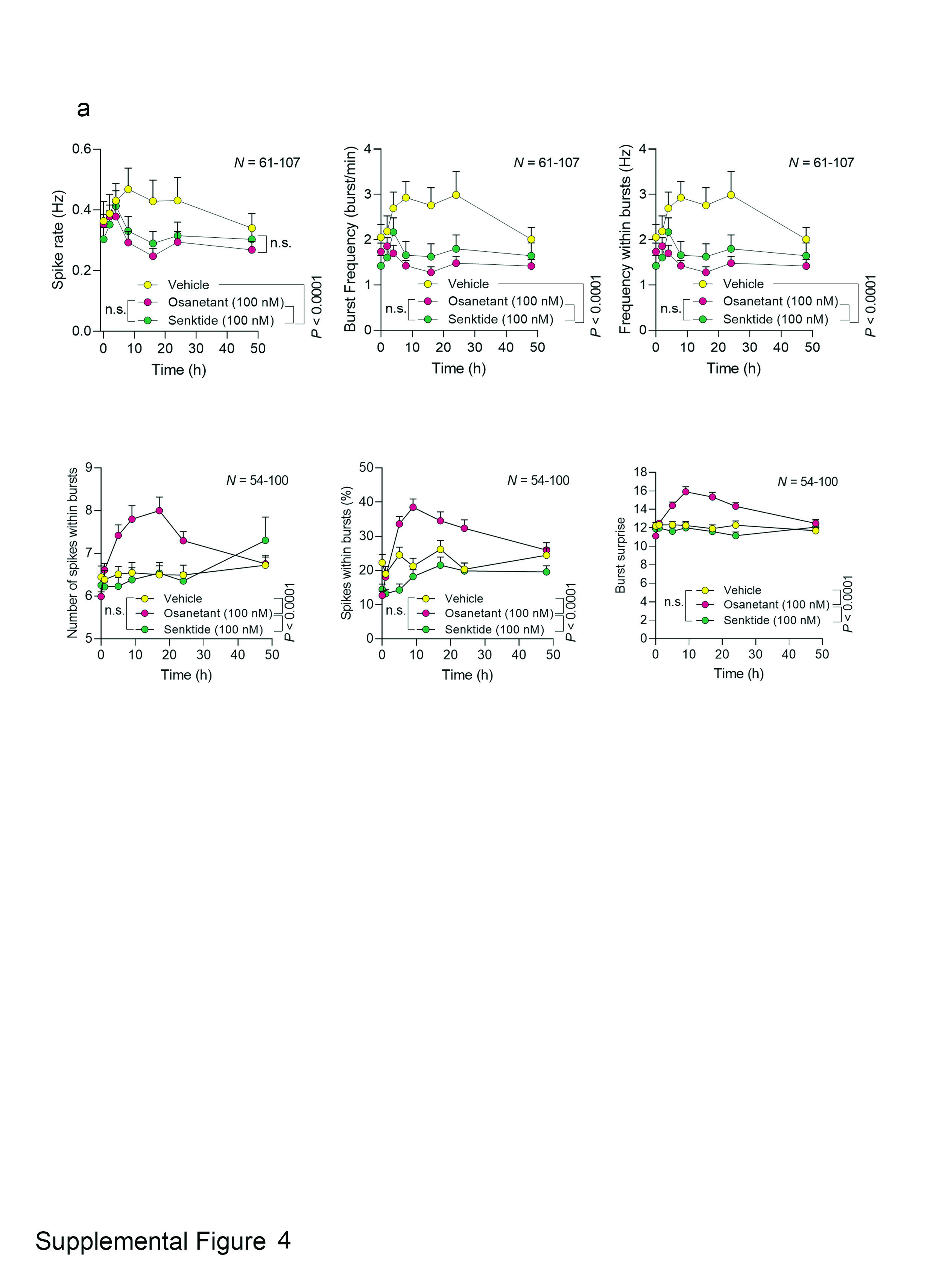

Supplement: Supplementary file 5 — Supplementary Figure 4. [file 41380_2023_2361_MOESM5_ESM.jpg]

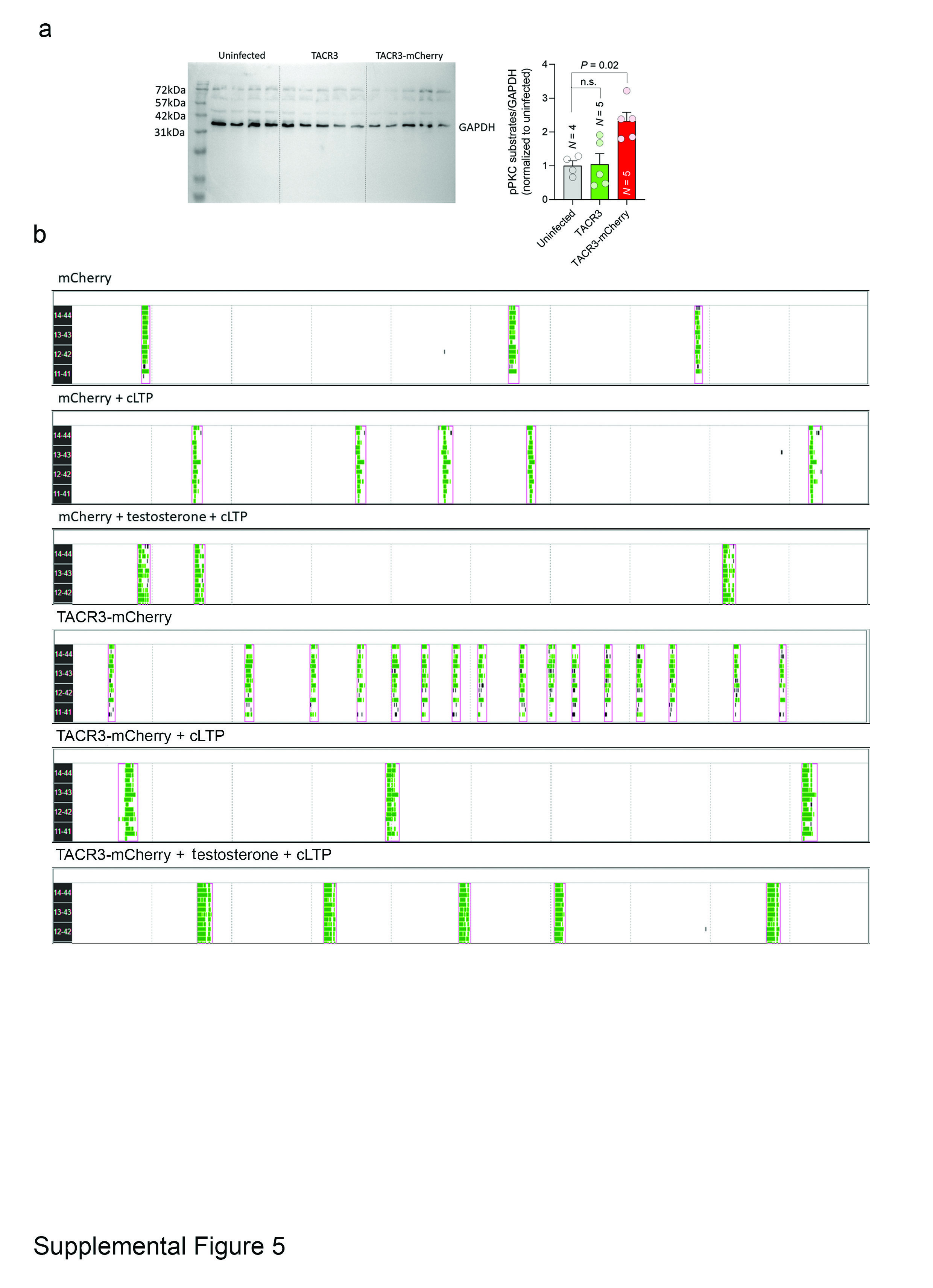

Supplement: Supplementary file 6 — Supplementary Figure 5. [file 41380_2023_2361_MOESM6_ESM.jpg]

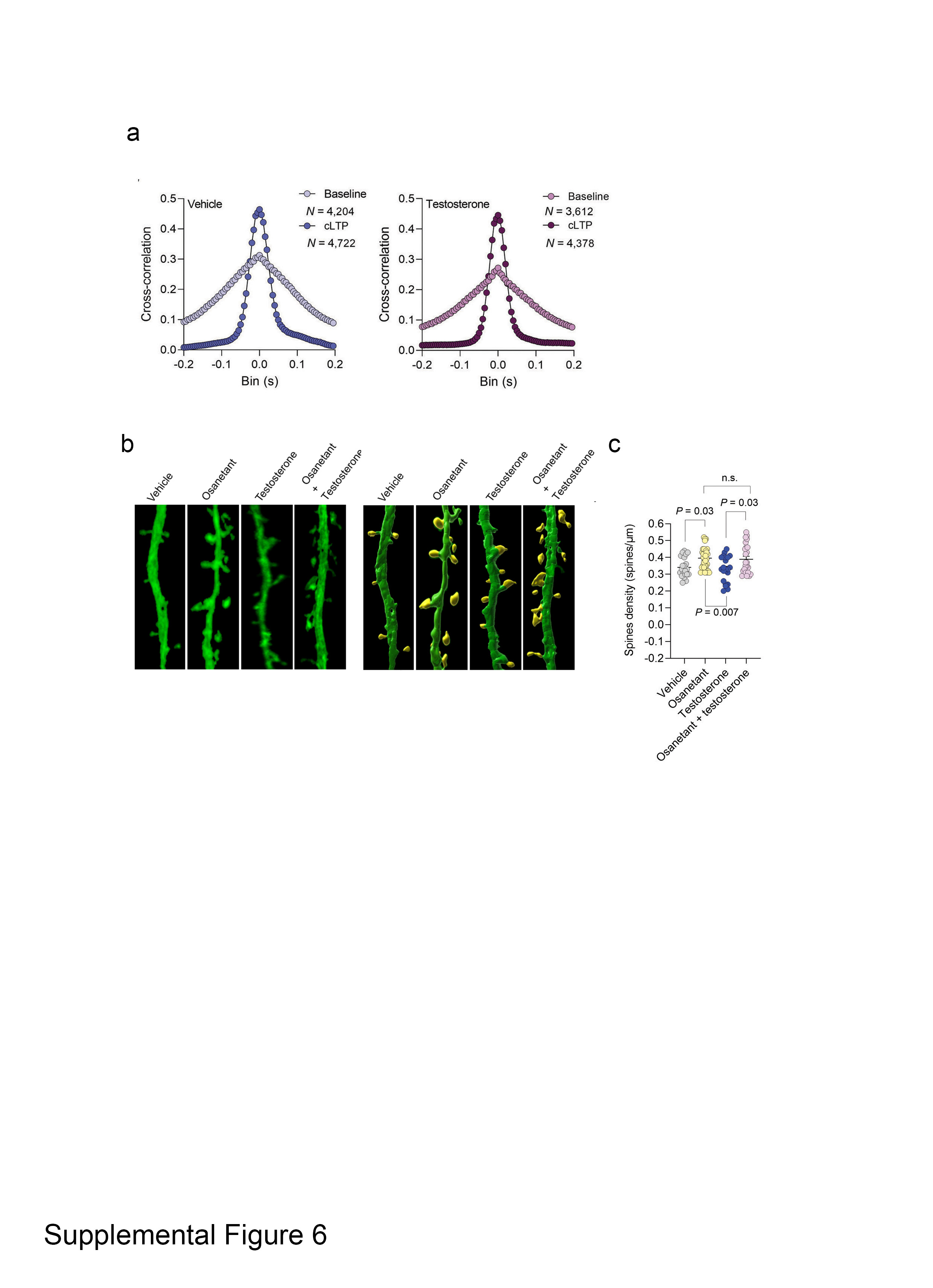

Supplement: Supplementary file 7 — Supplementary Figure 6. [file 41380_2023_2361_MOESM7_ESM.jpg]
